# Transcriptional changes in prostate of men on active surveillance after a 12-mo glucoraphanin-rich broccoli intervention—results from the Effect of Sulforaphane on prostate CAncer PrEvention (ESCAPE) randomized controlled trial

**DOI:** 10.1093/ajcn/nqz012

**Published:** 2019-04-15

**Authors:** Maria H Traka, Antonietta Melchini, Jack Coode-Bate, Omar Al Kadhi, Shikha Saha, Marianne Defernez, Perla Troncoso-Rey, Helen Kibblewhite, Carmel M O'Neill, Federico Bernuzzi, Laura Mythen, Jackie Hughes, Paul W Needs, Jack R Dainty, George M Savva, Robert D Mills, Richard Y Ball, Colin S Cooper, Richard F Mithen

**Affiliations:** 1 Quadram Institute Bioscience, Norwich, United Kingdom; 2 Department of Urology, Norfolk and Norwich University Hospitals NHS Foundation Trust, Norwich, United Kingdom; 3 Norfolk and Waveney Cellular Pathology Service, Norfolk and Norwich University Hospitals NHS Foundation Trust, Norwich, United Kingdom; 4 Norwich Medical School, University of East Anglia, Norwich, United Kingdom; 5 Liggins Institute, University of Auckland, New Zealand

**Keywords:** active surveillance, broccoli, dietary intervention, transcriptome, RNA sequencing, SMCSO, sulforaphane, prostate biopsy, cancer prevention

## Abstract

**Background:**

Epidemiological evidence suggests that consumption of cruciferous vegetables is associated with reduced risk of prostate cancer progression, largely attributed to the biological activity of glucosinolate degradation products, such as sulforaphane derived from glucoraphanin. Because there are few therapeutic interventions for men on active surveillance for prostate cancer to reduce the risk of cancer progression, dietary approaches are an appealing option for patients.

**Objective:**

We evaluated whether consumption of a glucoraphanin-rich broccoli soup for 1 y leads to changes in gene expression in prostate tissue of men with localized prostate cancer.

**Methods:**

Forty-nine men on active surveillance completed a 3-arm parallel randomized double-blinded intervention study for 12 mo and underwent transperineal template biopsy procedures and dietary assessment at the start and end of the study. Patients received a weekly 300 mL portion of soup made from a standard broccoli (control) or from 1 of 2 experimental broccoli genotypes with enhanced concentrations of glucoraphanin, delivering 3 and 7 times that of the control, respectively. Gene expression in tissues from each patient obtained before and after the dietary intervention was quantified by RNA sequencing followed by gene set enrichment analyses.

**Results:**

In the control arm, there were several hundred changes in gene expression in nonneoplastic tissue during the 12 mo. These were associated with an increase in expression of potentially oncogenic pathways including inflammation processes and epithelial–mesenchymal transition. Changes in gene expression and associated oncogenic pathways were attenuated in men on the glucoraphanin-rich broccoli soup in a dose-dependent manner. Although the study was not powered to assess clinical progression, an inverse association between consumption of cruciferous vegetables and cancer progression was observed.

**Conclusion:**

Consuming glucoraphanin-rich broccoli soup affected gene expression in the prostate of men on active surveillance, consistent with a reduction in the risk of cancer progression. This trial was registered at clinicaltrials.gov as NCT01950143.

## Introduction

The diagnosis of organ-confined prostate cancer has increased owing to routine prostate specific antigen (PSA) testing and an ageing population. Up to 48% of cases may exhibit clinical progression after subsequent examination, and a small proportion of these may become metastatic with associated poor prognosis (1, 2). However, owing to the risks associated with curative treatment, men with organ-confined prostate cancer may choose a program of “active surveillance,” in which radical treatment is delayed until there is evidence of cancer progression. Currently, there are no approved therapeutic interventions for men who have chosen a program of active surveillance that may reduce the risk of cancer progression.

Epidemiological studies have suggested a negative association between consumption of cruciferous vegetables and incidence or progression of prostate cancer (3–5). The protective activity has been associated with the biological activity of degradation products of glucosinolates, sulfur-containing glycosides that accumulate in these vegetables. When consumed, glucosinolates are degraded either due to the action of plant-derived thioglucosidases or, if these have been denatured as a result of cooking, by microbial activity in the colon (6). Glucosinolates with aliphatic or aromatic side chains produce isothiocyanates, such as sulforaphane derived from 4-methylsulphinylbutyl glucosinolate (glucoraphanin) that accumulates in broccoli. Glucosinolates with indole side chains produce indole-3-carbinol and associated metabolites (**Supplemental Figure 1**A and B) (7). These glucosinolate-derived metabolites exhibit a range of biological activity in model systems consistent with the protective effects of cruciferous vegetables (8). However, despite the large number of studies with model systems, there are few examples of human intervention studies with either biological or clinical endpoints to provide further evidence that diets rich in glucosinolates, glucoraphanin, or sulforaphane may prevent prostate cancer progression. Cruciferous vegetables also accumulate S-methyl cysteine sulfoxide (SMCSO) which, in an analogous manner to glucosinolates, degrades to bioactive metabolites (9, 10) (Supplemental Figure 1C).

We report a double-blinded randomized controlled trial to test the hypothesis that a diet rich in glucoraphanin, the glycosylated precursor of sulforaphane, would significantly modify gene and metabolite expression in the prostate of men on active surveillance for prostate cancer. We used broccoli genotypes specifically developed to have enhanced concentrations of glucoraphanin through the introgression of either 1 or 2 alleles of the *Myb28* transcription factor from the wild species *Brassica villosa* but with otherwise identical chemical profiles (11, 12). The primary outcome of the study was to detect changes in gene expression in response to glucoraphanin-rich diets through RNA sequencing from prostate biopsies, which were collected at the start of the study and after the 12-mo intervention. The secondary outcome was to analyze metabolites from these biopsies.

We analyzed sequential transperineal template prostate biopsy samples from prostate cancer patients immediately before (T0) and after (T12) a 12-mo intervention with a broccoli soup made from 1 of the 3 broccoli genotypes, and reported paired analyses of global gene expression, gene set enrichment analyses (GSEAs), and metabolite profiles (i.e., at T0 and T12) for each of the volunteers. As several studies have reported interactions between diet and Glutathione S-transferase mu1 (*GSTM1*) genotype, we also investigated whether *GSTM1* genotype may affect the response to the dietary intervention. Finally, we quantified the correlation between the intake of individual food components and the clinical parameters of the patient cohort.

## Methods

### Ethics

The study (NCT01950143) was approved by the Quadram Institute Bioscience Human Research Governance Committee and by the National Research Ethics Service (Research Ethics Committee ref: 13/EE/0110).

### Study design

Effect of Sulforaphane on prostate CAncer PrEvention (ESCAPE) was a randomized, double-blinded 3-arm parallel intervention recruiting men aged 18–80 y with a BMI between 19.5 and 35 kg/m^2^. The men had a diagnosis of low-risk prostate cancer (PSA < 10 ng/mL, Gleason grade 6; T category T1 or T2) or intermediate-risk prostate cancer (PSA 10–20 ng/mL, Gleason 7, including selected 4 + 3 cases that made informed decisions against radical treatment; T category T1 or T2) and were undergoing active surveillance. Complete eligibility and exclusion criteria are detailed in **Supplemental Table 1**. The primary outcome was gene expression of prostate tissue obtained before and after a dietary intervention and the secondary outcome was changes in metabolites. The study was powered based upon data obtained from a previous pilot study (13). The number of volunteers necessary to report statistically significant changes in gene expression was calculated by 2 methods: firstly, by using the “Sample Size for Microarray Experiments” tool developed by the Section of Bioinformatics of the University of Texas MD Anderson Cancer Center (https://biostatistics.mdanderson.org/MicroarraySampleSize/); and secondly, by reported calculations based on previously published data (14). We estimated that 26 subjects in each of the 3 dietary groups (78 in total) were required to detect 1.5-fold differences with a significant difference (*P* < 0.02) between any 2 of the 3 dietary groups, with a power of 80% and an SD of 0.66 (based on a log2 scale of gene intensity measurements). However, the accrual rate was below that anticipated and recruitment was stopped before reaching the target sample size goal in order to complete the study within the scheduled date of closure (October, 2016). Patients (*n* = 61) were recruited through the Urology Department of the Norfolk and Norwich University Hospitals NHS Foundation Trust from October, 2013 to October, 2015.

Study patients were randomly allocated to 1 of 3 dietary arms in which they were required to consume 1 portion of broccoli soup (300 mL) per week as part of their normal diet for 12 mo, with an option to continue the intervention for a further 12 mo. For an optional extension (12–24 mo), patients underwent regular blood analyses, as described below, but no additional study biopsies were collected. Block randomization (www.randomization.com) and blinding were performed by an individual who was not part of the study team. The soups were manufactured by Bakkavor from the commercial cultivar Iron (soup X, genotype *Myb28* B/B), the cultivar Beneforte (soup Y, *Myb28* B/V, in which V represents an introgressed *Myb28* allele from *B. villosa*), or a noncommercial hybrid cultivar (soup Z, *Myb28* V/V). Three-hundred-milliliter portions of soups manufactured from these 3 genotypes contained 72 ± 2.8 (soup X), 214 ± 7.3 (soup Y), and 492 ± 3.2 (soup Z) μmol 4-methylsulphinylbutyl glucosinolate (glucoraphanin). A previous study reported that these different soup products resulted in contrasting concentrations of sulforaphane in the systemic circulation (12). One weekly portion of soup X was assigned as the control arm, because this soup was manufactured from a commercial cultivar of broccoli and could be expected to be part of a normal diet. This soup provided the lowest concentration of glucoraphanin, which, and on the basis of epidemiological studies (3), would be considered beneath the threshold required for a reduction in cancer progression.

Patients underwent 2 transperineal template biopsy (TTB) procedures, 1 at the start of the intervention (T0) and 1 after 12 mo (T12). Of the 24–56 TTB cores per patient obtained at each TTB, several were individually reserved either in RNAlater for RNA sequencing or in extraction solvent (80% HPLC grade methanol:20% water) for targeted and nontargeted metabolite analyses. Two further cores were snap frozen, and the remainder underwent routine histopathological examination. The tissue was examined by a single consultant histopathologist with a special interest in prostate pathology to reduce interobserver error, a potential hazard in diagnosing and grading prostate cancer (15). After histopathology, cores were selected for RNA sequencing analyses that were adjacent to cores that did not contain cancer.

### Dietary analyses

Patients completed a comprehensive 7-d diet diary immediately before the study to assess their habitual diet, and subsequently at 6 and 12 mo. Diet data were analyzed through DietPlan6 (Forestfield Software Ltd, UK) and combined with additional data on the chemical composition of cruciferous and alliaceous vegetables, obtained from analyses of vegetables purchased in retail outlets in the localities of the volunteers, as previously described (16).

### Gene expression analyses by RNA sequencing

The primary outcome of the study was to detect changes in gene expression in response to the dietary intervention through RNA sequencing, from prostate biopsies collected at the start of the study and at 12 mo, i.e., after the intervention. Histology of the directly adjacent region confirmed that the prostate biopsies used for RNA sequencing were unlikely to contain neoplastic tissue. Cores of between 3 and 10 mg from each patient were homogenized with a QIAGEN TissueRuptor before total RNA was extracted with the QIAGEN RNeasy Mini kit. The resulting RNA was quality checked with an Agilent Bioanalyzer and samples with RIN values >7 were further processed. Samples were ribodepleted with the Ribo-Zero Magnetic Gold rRNA Removal Kit (Illumina) before constructing Illumina barcoded TruSeq RNA libraries. Sequencing of 98 libraries was performed on an Illumina HiSeq 2500/2000 in high-output mode using 125-bp paired-end reads, generating 50–70 million reads/library. RNA-seq reads were first processed by removing Illumina adapters using *Trim Galore!* version 0.4.2 (Babraham Bioinformatics) and reads with Phred quality of basecalls >20 and with a length of >60 bp were carried forward. *SortMeRNA* version 2.1 (17) was used to filter any remaining ribosomal RNA from the adaptor and quality trimmed reads.

Reads were analyzed using the *HISAT2-StringTie* pipeline (18) aligned to the Ensembl GRCh38.89 reference genome (*HISAT2* version 2.0.5 and *StringTie* version 1.3.3), and gene counts were exported into *edgeR* in *R Bioconductor* (19). One patient was removed from all analyses because he underwent prostatectomy at 12 mo, rather than TTB, leaving a total of 48 patients (96 libraries). The complete bioinformatics pipeline, differential gene expression (DGE) analyses, and statistical analysis are available as a GitHub repository (https://github.com/quadram-institute-bioscience/ESCAPE_RNAseq_analysis).

In addition, we conducted a query on publicly available RNA sequencing data that were generated using the same Illumina HiSeq 2000/2500 technology as our samples, had ≥100-bp paired reads, and contained benign as well as primary cancer samples. We identified accession GSE80609 in Gene Expression Omnibus (https://www.ncbi.nlm.nih.gov/geo) and downloaded the raw reads (.fastq) before analyzing them with the same pipeline as our samples, described above. When ESCAPE and GSE80609 samples were put together in *edgeR* we generated multidimensional scaling (MDS) plots for the second and third dimensions, because the first dimension was clearly only differentiating the 2 separate studies (data not shown).

RNA sequencing data from the ESCAPE study have been deposited in ArrayExpress (accession E-MTAB-6525).

### Metabolomics analyses

Desiccated methanol extracts, derived from 24-h incubation of 1 prostate biopsy per patient, were sent to Metabolon Inc. to undergo ultra-HPLC–mass spectroscopy (MS) and gas chromatography–MS with a high resolution accurate mass (HRAM) platform as previously described (20) (www.metabolon.com). A total of 448 metabolites were semiquantified on the basis of ion count within several biologically relevant classes (amino acids, carbohydrates, vitamins, lipids, nucleotides, peptides, tricarboxylic acid cycle, and xenobiotics). Histology of the core after incubation confirmed the absence of cancerous foci.

### Blood analyses

Biomarkers of liver and kidney function and full blood count were quantified at T0 and T12 to ensure the glucoraphanin-rich soup had no toxic effects. Fasting blood glucose, PSA, and serum lipid profile (cholesterol, HDL cholesterol, LDL cholesterol, triglyceride) were quantified at 3-mo intervals, up to 24 mo from the start of the study. *GSTM1* genotype was quantified as previously described (13).

### Statistical analyses

#### Analyses of clinical characteristics

The difference between clinical parameters (age, BMI, PSA) between the 3 different groups at the start and at the end of the study was assessed either by using ANOVA and correcting for multiple testing by Tukey's multiple correction test, or by Kruskal–Wallis test corrected for multiple testing by Dunn's, where appropriate.

#### DGE analyses

Paired DGE analysis after calculation of normalized gene counts was undertaken in *limma* after *voom* transformation (21). Adjustment for multiple testing was performed using the Benjamini–Hochberg false discovery rate (FDR) method. MDS plots generated in *EdgeR* were used to determine the variation within different groups at the start and the end of the study. Statistical significance of unadjusted and FDR-adjusted *P* values was reported for different thresholds.

#### Functional analyses

Functional analyses of paired DGE were undertaken by the GSEA software (22) using the Hallmark gene sets (50 gene sets in total) within the available Molecular Signatures Database (MSigDB, version 6.1). DGE lists were ranked according to their *P* value, modified using the rank–rank hypergeometric overlap (RRHO) algorithm (23). Modified *P* values were calculated as the signed log_10_-transformed *P* value of the paired log fold change over 12 mo for each dietary arm, with the sign denoting the direction of the change: positive for upregulated over time, and negative for downregulated over time. By using the RRHO method we explored the functional consequences of the paired changes in gene expression without being constrained by a given statistical threshold. The ranked DGE list was then submitted to GSEA and statistical significance of enriched pathways was set at an FDR-adjusted *P* value <0.05. Normalized enrichment scores for each individual pathway and their associated FDR-adjusted *P* value for each diet were reported with and without stratification by *GSTM1* genotype. An MDS plot was generated in *EdgeR* to determine the variation in normalized enrichment scores for the different dietary groups stratified by *GSTM1* genotype.

#### Metabolomics analyses

Paired Student's *t* tests were undertaken for each individual metabolite within each dietary arm. Adjustment for multiple testing was performed using the Benjamini–Hochberg FDR method. Comparisons of log_2_-fold changes for each metabolite between dietary arms were made by unpaired *t* tests with FDR correction.

#### Exploratory association with clinical outcomes

Individual dietary components, calculated from the diet diaries of the patients reported at the start and the end of the study, were tested for association with histological and blood markers. This exploratory analysis was undertaken in the R environment (R Foundation) using Pearson correlations. Gleason scores were adjusted to be no lower than any previous biopsy, to correct for the undetected cancers in this cohort, and subsequently were converted to risk groups according to the WHO grade group system (24), to allow differentiation between Gleason 7 scores (3 + 4 or 4 + 3), which occupy different grade groups under the WHO system.

## Results

### Clinical characteristics and dietary assessment of subjects

**FIGURE 1:**
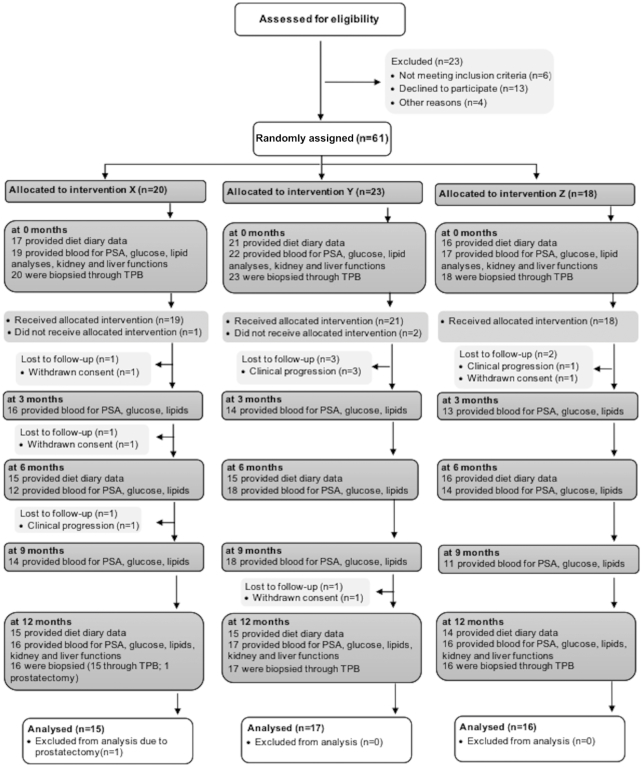
Consolidated Standards of Reporting Trials (CONSORT) flow diagram of patients on surveillance receiving 1 of the 3 dietary interventions over 12 mo and the schedule of biopsies and analyses. PSA, prostate-specific antigen; TPB, trans perineal biopsy.

Sixty-one men on active surveillance were randomly assigned to 1 of the 3 dietary intervention arms and 49 completed the study (**Figure 1**). Of the 12 volunteers who did not complete the study, 5 exhibited clinical progression before the start of the dietary intervention after their first TTB, 4 withdrew consent during the study for unknown reasons, and 3 did not undergo a second TTB for either clinical or personal reasons. There were no significant differences in age, BMI, frequency of *GSTM1* null genotypes, or PSA between the 3 groups at the start or the end of the study (**Table 1**). Similarly, there were no significant changes in biomarkers of kidney and liver function and metabolism over time or differences between the 3 arms of the study, during the 12-mo study (Table 1; **Supplemental Table 2**). Fasting blood glucose concentrations showed an initial fall over the first 6 mo of the study, and then a further fall between 12 and 18 mo (**Supplemental Figure 2**).

**TABLE 1:** Patient characteristics^1^

	Soup X (control; low GR)	Soup Y (intermediate GR)	Soup Z (high GR)
*n*	15	17	16
*n* of *GSTM1* (−/−)	9	10	12
Age,^2^ y	68 ± 5	66 ± 6	66 ± 6
BMI,^2^ kg/m^2^	26.7 ± 3.1	27.6 ± 3.4	27.7 ± 2.2
Days from initial diagnosis^2^	309 ± 232	312 ± 356	327 ± 292
At diagnosis			
PSA, µg/L^3,4^	7.7 (5.9–7.9)	6.8 (5.6–8.6)	7 (5.0–9.3)
Gleason score,^5^*n*			
3 + 3	10	10	9
3 + 4	4	5	6
4 + 3	1	2	0
At 0 mo			
PSA, µg/L^3^	7.9 (5.9–12.0)	7.6 (4.9–9.4)	5.8 (4.4–8.7)
PSA density^3^	0.10 (0.08–0.18)	0.13 (0.09–0.18)	0.10 (0.08–0.16)
Gleason score			
3 + 3	5	7	1
3 + 4	7	5	10
4 + 3	0	0	0
Undetected	3	5	5
Core ratio^3^	7.9 (3.0–14.3)	3.7 (0–9.4)	11.6 (0–16.1)
At 12 mo			
PSA, µg/L^3^	9.4 (6.6–10.4)	7.3 (6.9–10.4)	7.5 (5.6–9.3)
PSA density^3^	0.13 (0.10–0.02)	0.12 (0.08–0.17)	0.11 (0.08–0.14)
Gleason score			
3 + 3	2	5	2
3 + 4	9	4	10
4 + 3	0	1	3
4 + 4	1	0	0
Undetected	3	7	1
Core ratio^3^	7.7 (4.2–21.7)	4.4 (0–16.6)	13 (6.4–17.5)

^1^GR, glucoraphanin; *GSTM1*, Glutathione S-transferase M1; PSA, prostate specific antigen.

^2^Nonsignificant difference (i.e., all *P* values >0.5) between diets as determined by ANOVA adjusted by Tukey's multiple correction test. Data shown are mean ± SD.

^3^Nonsignificant difference (i.e., all *P* values >0.2) between diets as determined from the Kruskal–Wallis test adjusted by Dunn's multiple correction test. Data shown are median (IQR).

^4^PSA at diagnosis missing for 3 patients on Diet X, 1 on Diet Y, and 1 on Diet Z.

^5^Gleason score at diagnosis missing for 1 patient on Diet Z.

At baseline, there were no differences in the habitual diet of the volunteers between the 3 arms, and there were no changes during the 12-mo intervention period with the exception of glucoraphanin intake which, as expected, was significantly different between the 3 arms owing to the provision of the broccoli soups (**Supplemental Table 3**).

### Gene expression profiles of nonneoplastic prostate biopsies

**FIGURE 2:**
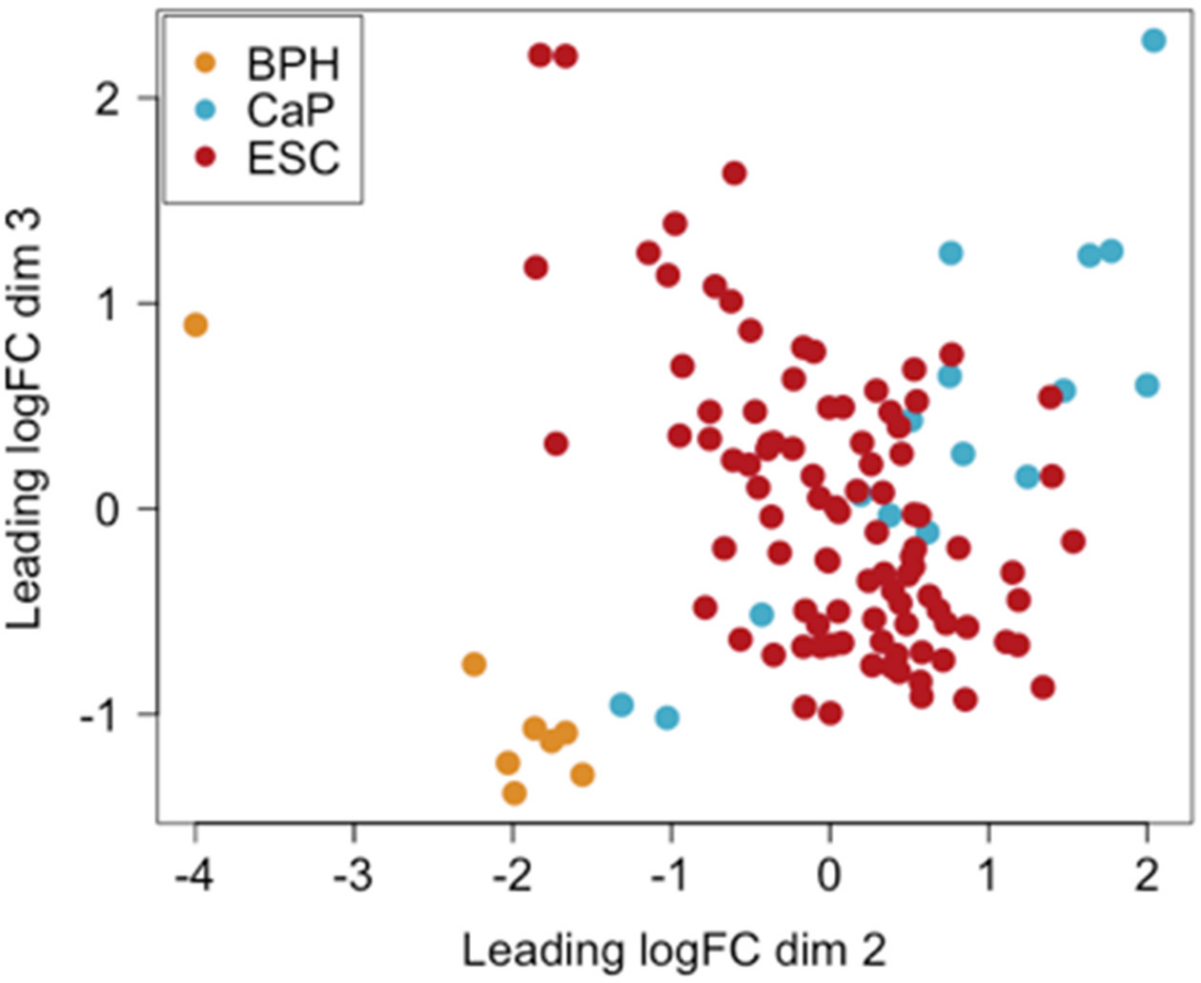
Multidimensional scaling plot of the ESCAPE cohort prostate biopsies (ESC) alongside publicly available (GSE80609) BPH and CaP samples. BPH, benign prostatic hyperplasia; CaP, primary prostate cancer; ESCAPE, Effect of Sulforaphane on prostate CAncer PrEvention; FC, fold change.

We first compared the transcriptional prostate signature of our cohort with that from a previous study that compared transcriptional profiles from benign prostate hyperplasia and primary prostate cancer (25). Apparent nonneoplastic biopsies from the ESCAPE patients were intermediate between the BPH samples and the prostate cancer samples, although with some overlap with the cancer samples (**Figure 2**). This indicates that transcriptional changes may be occurring across the whole prostate of patients on active surveillance, with some similarities with those occurring in the cancer lesions themselves, consistent with a “field effect.”

In order to determine whether the transcriptional profiles of the 3 groups at the start or the end of the study differed despite the random group assignment, we used MDS plots to assess variability and found no evidence of outliers, either in single patients or in diet groups (**Supplemental Figure 3**A and B).

### Paired DGE analyses and GSEAs

We investigated the changes in gene expression that occurred within each arm over time. In the control group (i.e., those that received a single portion of broccoli soup made from standard broccoli, genotype *Myb28* B/B, soup X) we found significant changes in gene transcription (FDR-adjusted *P* < 0.1, **Table 2**).

**TABLE 2:** Number of genes changed over time with the different diets^1^

	Soup X (*n* = 15) (control; low GR)	Soup Y (*n* = 17) (intermediate GR)	Soup Z (*n* = 16) (high GR)
FDR-adjusted *P* value^2^			
<0.05	7 (4↑, 3↓)	0	0
<0.1	96 (58↑, 38↓)	0	0
*P* value^3^			
<0.001	154 (82↑, 72↓)	26 (20↑, 6↓)	12 (8↑, 4↓)
<0.01	980 (520↑, 460↓)	331 (224↑, 107↓)	83 (46↑, 37↓)
<0.05	2796 (1460↑, 1336↓)	1359 (783↑, 576↓)	502 (277↑, 225↓)

^1^FDR, false discovery rate; GR, glucoraphanin. ↑ indicate increase in gene expression. ↓ indicate decrease in gene expression.

^2^Paired *t* tests, adjusted for multiple testing correction by Benjamini–Hochberg.

^3^Student's paired *t* tests, unadjusted for multiple testing correction.

GSEA, with the use of the RRHO method that takes into account all the genes ranked by *P* value and fold change, identified significant enrichment of pathways associated with the risk of carcinogenesis (FDR-adjusted *P* < 0.05, **Table 3**), including inflammatory response (**Supplemental Figure 4**) and epithelial–mesenchymal transition (**Supplemental Figure 5**). Among the significantly enriched pathways were also those associated with androgen response (FDR-adjusted *P* < 0.001), angiogenesis (FDR-adjusted *P* < 0.001), and apoptosis (FDR-adjusted *P* < 0.002; Table 3).

**TABLE 3:** GSEA of paired changes over time for the control arm (soup X, low GR) and the experimental arms (soup Y, intermediate GR; soup Z, high GR)^1^

	Soup X (control; low GR)			Soup Y (intermediate GR)		Soup Z (high GR)	
MSigDb pathway	SIZE	NES	FDR *P* value^2^	NES	FDR *P* value^2^	NES	FDR *P* value^2^
TNFA signaling via NFKB	167	2.65^3^	0 ^3^	2.89 ^3^	0 ^3^	−1.33	0.121
Epithelial–mesenchymal transition	176	2.70 ^3^	0 ^3^	2.30 ^3^	0 ^3^	−1.03	0.558
Hypoxia	164	1.95 ^3^	0 ^3^	1.88 ^3^	0 ^3^	−0.97	0.655
Inflammatory response	152	1.95 ^3^	0 ^3^	2.36 ^3^	0 ^3^	0.95	1
TGF β signaling	46	2.03 ^3^	0 ^3^	1.58 ^3^	0.007 ^3^	0.82	1
Protein secretion	91	−1.99 ^3^	0 ^3^	−1.56 ^3^	0.012 ^3^	−1.36	0.095
Androgen response	93	−2.13 ^3^	0 ^3^	−1.62 ^3^	0.014 ^3^	−1.45	0.055
Myogenesis	173	2.01 ^3^	0 ^3^	1.51 ^3^	0.018 ^3^	−2.30 ^3^	0 ^3^
UV response DN	133	2.11 ^3^	0 ^3^	1.29	0.090	−0.77	0.953
Angiogenesis	30	1.89 ^3^	0.001 ^3^	2.06 ^3^	0 ^3^	−1.61 ^3^	0.016 ^3^
IL2 STAT5 signaling	154	1.84 ^3^	0.001 ^3^	2.21 ^3^	0 ^3^	−1.25	0.191
Coagulation	89	1.76 ^3^	0.001 ^3^	2.08 ^3^	0 ^3^	−0.85	0.924
Interferon-γ response	162	1.81 ^3^	0.001 ^3^	2.47 ^3^	0 ^3^	0.77	0.931
KRAS signaling UP	157	1.81 ^3^	0.001 ^3^	1.88 ^3^	0 ^3^	−0.81	0.957
Apoptosis	140	1.71 ^3^	0.002 ^3^	1.99 ^3^	0 ^3^	−1.19	0.259
Notch signaling	29	1.68 ^3^	0.003 ^3^	1.08	0.344	1.10	0.851
Fatty acid metabolism	127	−1.76 ^3^	0.003 ^3^	0.88	0.765	−1.64 ^3^	0.016 ^3^
IL6 JAK STAT3 signaling	61	1.63 ^3^	0.004 ^3^	2.13 ^3^	0 ^3^	−0.66	0.989
Unfolded protein response	98	−1.68 ^3^	0.004 ^3^	1.49 ^3^	0.020 ^3^	−1.58 ^3^	0.018 ^3^
Cholesterol homeostasis	63	−1.72 ^3^	0.004 ^3^	0.73	0.956	−1.43	0.062
Apical junction	163	1.60 ^3^	0.005 ^3^	2.04 ^3^	0 ^3^	−1.23	0.206
Peroxisome	82	−1.63 ^3^	0.008 ^3^	0.99	0.504	−1.02	0.567
Complement	144	1.44 ^3^	0.025 ^3^	1.96 ^3^	0 ^3^	0.97	1
Mitotic spindle	171	1.44 ^3^	0.025 ^3^	1.23	0.150	1.24	0.482
P53 pathway	171	1.41 ^3^	0.033 ^3^	2.05 ^3^	0 ^3^	−0.97	0.665
Allograft rejection	146	1.39 ^3^	0.035 ^3^	2.13 ^3^	0 ^3^	1.28	0.697
MYC targets V1	178	−1.44	0.051	1.48 ^3^	0.021 ^3^	−2.11 ^3^	0 ^3^
MTORC1 signaling	179	−1.42	0.054	1.35	0.066	−1.50 ^3^	0.039 ^3^
Interferon-α response	81	1.27	0.091	2.00 ^3^	0	−0.78	0.974
Estrogen response early	167	1.19	0.159	1.99 ^3^	0	−1.24	0.202
DNA repair	117	−1.26	0.187	1.40 ^3^	0.044 ^3^	−1.13	0.355
Hedgehog signaling	30	1.11	0.260	1.66 ^3^	0.003 ^3^	−0.95	0.651
UV response UP	128	1.10	0.266	1.73 ^3^	0.001 ^3^	−1.71 ^3^	0.009 ^3^
Reactive oxygen species	42	−1.14	0.302	1.32	0.077	−1.63 ^3^	0.014 ^3^
MYC targets V2	51	−1.14	0.323	1.82 ^3^	0 ^3^	−1.71 ^3^	0.011 ^3^
Xenobiotic metabolism	139	−1.09	0.356	1.48 ^3^	0.020 ^3^	−1.65 ^3^	0.016 ^3^
Oxidative phosphorylation	174	−1.08	0.366	0.79	0.907	−2.16 ^3^	0 ^3^
Estrogen response late	162	−1.01	0.505	1.88 ^3^	0 ^3^	−1.28	0.163
Adipogenesis	164	−0.93	0.736	1.10	0.328	−1.86 ^3^	0.002^3^
E2F targets	147	−0.86	0.828	1.42 ^3^	0.036 ^3^	0.93	1

^1^FDR, false discovery rate [as described in (22)]; E2F, E2 Factor; GR, glucoraphanin; GSEA, gene set enrichment analysis; IL6, interleukin 6; JAK, Janus Kinase; KRAS, V-Ki-Ras2 Kirsten Rat Sarcoma 2 Viral Oncogene Homolog; MSigDb, Molecular Signature Database; MTORC1, mammalian target of rapamycin complex 1; MYC, myelocytomatosis; NES, normalized enrichment score; NFKB, nuclear factor kappa B1; STAT, signal transducer and activator of transcription; TGF, transforming growth factor; TNFA, tumor necrosis factor a; UV, ultra violet. SIZE refers to the numbers of genes in the pathway. UV response DN refers to genes that are down regulated by UV radiation. UV response UP refers to genes that are up regulated by UV radiation.

^2^GSEA by GSEA software version 3.0 on all genes ranked by the significance of fold change (see Methods section for details; http://software.broadinstitute.org/gsea).

^3^Only pathways significant at FDR-adjusted *P* < 0.05 in ≥1 of the 3 diets are shown.

**FIGURE 3:**
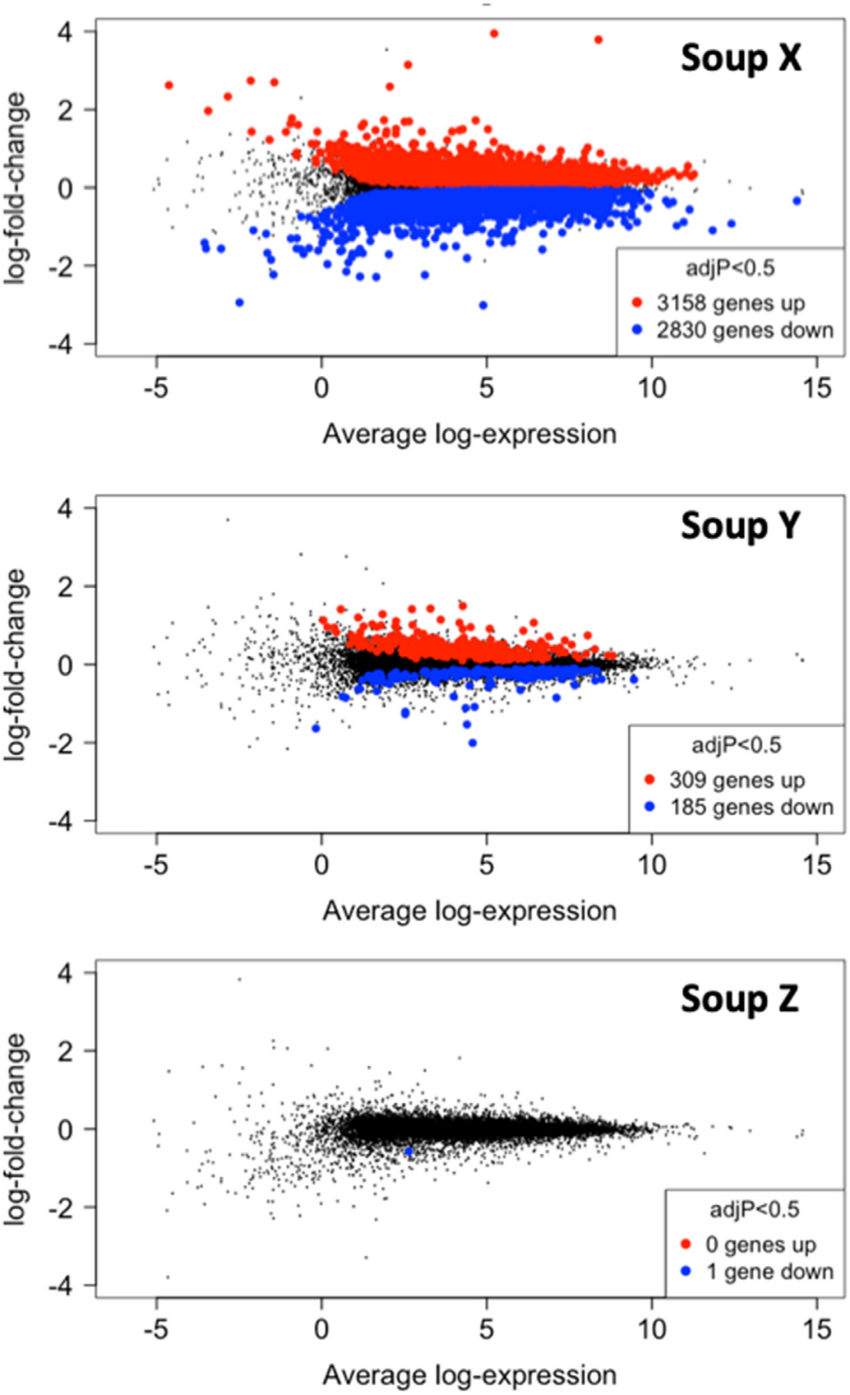
Volcano plots of differentially expressed genes over time. Highlighted are the genes that are significantly upregulated or downregulated over time in each dietary arm, colored in red or blue, respectively. Even at a low statistical threshold we do not observe any changes in gene expression with the high-glucoraphanin soup (Soup Z) (false discovery rate–adjusted *P* value <0.5, paired *t* tests adjusted by Benjamini–Hochberg for multiple testing correction). adjP refers to a probability value that has been adjusted for multiple testing with the use of Benjamini-Hochberg false discovery rate method.

When the intake of glucoraphanin was enhanced through the use of broccoli soup with genotype *Myb28* V/B (soup Y) or genotype *Myb28* V/V (soup Z), the extent of change in gene expression over time was suppressed, even at a low statistical threshold (**Figure 3**). Interestingly, men who consumed the highest amount of glucoraphanin (soup Z) had only 1 gene that significantly changed in expression over the 12-mo period (FDR-adjusted *P* < 0.5), in contrast to the multiple changes in the control arm (soup X) or the more modest changes in the intermediate glucoraphanin arm (soup Y; Table 2, Figure 3).

In the same manner as we did for the control arm, we explored the functional pathways enriched by soups Y and Z. GSEAs for soup Y were largely similar to those of the control soup. However, soup Z contrasted markedly with the control soup, by a lack of significant enrichment for the majority of pathways, including inflammatory response and epithelial–mesenchymal transition (Table 3). Moreover, in contrast to control soup X, there was significant enrichment of downregulated genes for reactive oxygen species and xenobiotic metabolism pathways by soup Z (Table 3).

### Analyses with *GSTM1* stratification

Analyses of the paired gene expression in the 3 dietary arms stratified by *GSTM1* null and non-nulls suggested that within intervention arms receiving soups X and Y the changes in gene expression were restricted to *GSTM1* non nulls (FDR-adjusted *P* < 0.1, **Supplemental Table 4**). GSEAs for the *GSTM1* nulls and non nulls were very similar in soup X, in terms of direction and magnitude of change of pathways, but exhibited some divergence in soups Y and Z indicative of a possible diet–gene interaction (**Supplemental Table 5, Supplemental Figure 6**).

### Effect of intervention on nuclear factor (erythroid-derived 2)-like 2–regulated genes

Sulforaphane, which would have been derived from the glucoraphanin delivered by the 3 different soups, is a potent inducer of nuclear factor (erythroid-derived 2)-like 2 (NRF2)-regulated genes. We therefore extracted from the RNA sequencing data the expression of previously defined NRF2-target genes (26) (**Supplemental Table 6**). There was no evidence of a change in expression of any NRF2-regulated genes between the start and end of the dietary intervention (Benjamini–Hochberg FDR-adjusted *P* value <0.1).

### Metabolomics analyses

Paired analyses of metabolites from tissue biopsies did not identify any significant changes in metabolites within any of the 3 dietary intervention arms. There was also no evidence for differences in fold changes in metabolites between dietary arms (data not shown).

### Exploratory analysis with clinical parameters of prostate cancer progression

**FIGURE 4:**
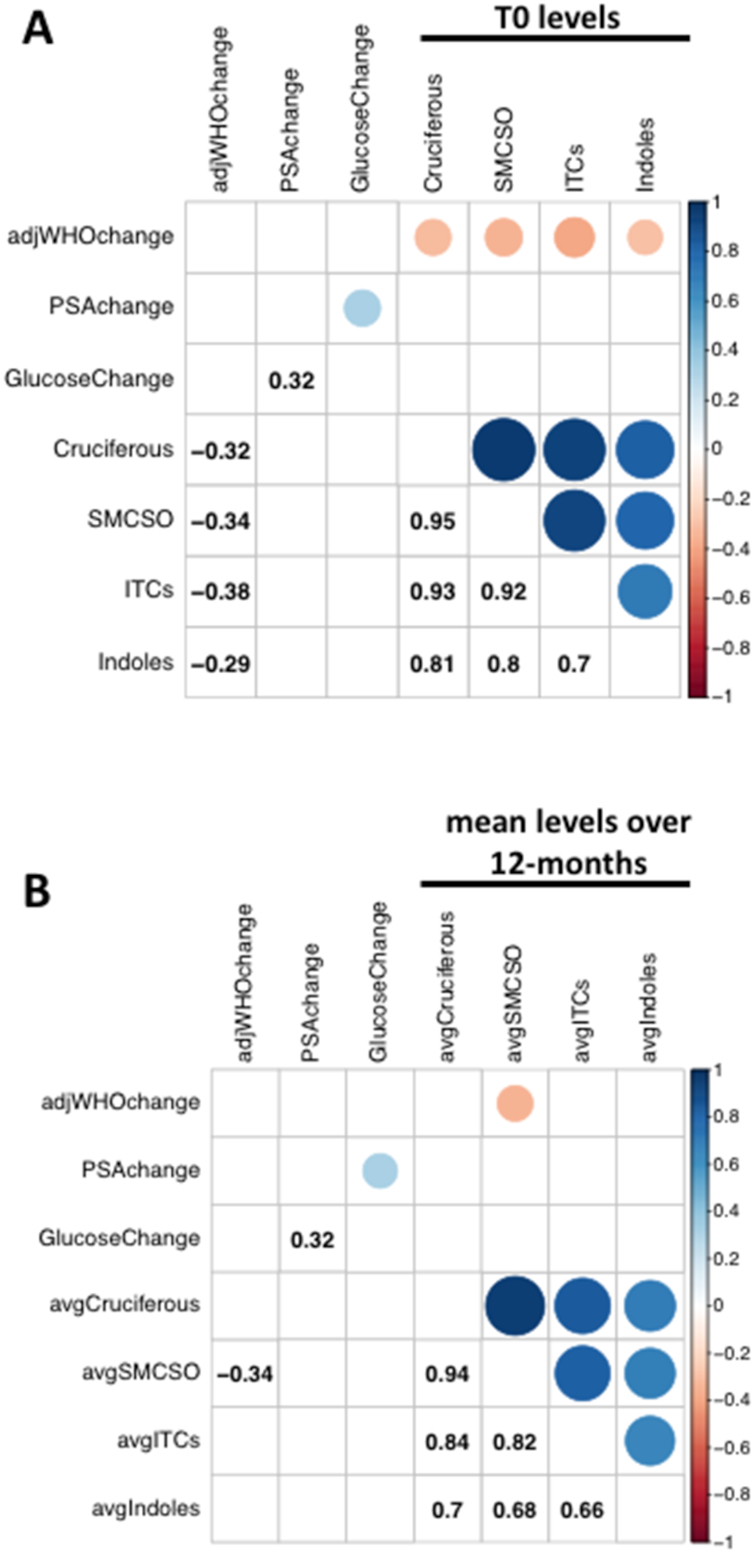
Analysis of dietary components. (A) Correlation matrix of the change in blood and histological markers over 12 mo, and the baseline concentrations of cruciferous vegetables and sulfur-metabolites. (B) Correlation matrix of the change in blood and histological markers over 12 mo, and the mean concentrations of cruciferous vegetables and sulfur-metabolites over the same period. Color denotes the direction of the Pearson correlation and dot size denotes the significance; only significant correlations (*P* < 0.05) are shown; numbers denote the Pearson correlation. ITC, isothiocyanate; PSA, prostate specific antigen; SMCSO, S-methyl cysteine sulfoxide. adjWHO grade refers to a WHO grade that has been adjusted to be no lower than that recorded from a previous biopsy, as described in materials and methods.

Ten out of 48 patients (28%) exhibited an increase in their cancer grade over the 12 mo of the study. Although our study was not powered to assess a clinical endpoint of prostate cancer progression, we undertook exploratory analysis and observed that the dietary intake of cruciferous vegetables at the start of the study (T0) was significantly inversely correlated with the change in WHO grade over the 12-mo study period (**Figure 4**A). This association was still apparent at T12, but not significant at *P* < 0.05 (data not shown). When we averaged the dietary intakes over 12 mo, the most significant inverse relation was between consumption of SMCSO and increase in WHO grade (Figure 4B).

## Discussion

The primary aim of the study was to test the hypothesis that enhancing glucoraphanin in the diet would result in changes in gene expression in prostate tissue of men on active surveillance that are consistent with reduction in the risk of cancer incidence or progression.

One of the challenges in seeking evidence for the protective effects of dietary components within a complex food matrix is experimental design. To test the effect of glucoraphanin within broccoli, we used 3 broccoli genotypes with contrasting glucoraphanin contents due to their *Myb28* genotype, enabling a randomized double-blinded dietary intervention study.

We analyzed changes in gene expression from sequential prostate tissue biopsies of the same individuals and found that in the control/placebo arm (i.e., commercially available broccoli) several hundred changes in gene expression occurred within the 12-mo period (Table 2, Figure 3). Subsequent GSEA indicated that the tissue within the control arm was likely to be at risk of carcinogenesis, with increased expression of several pathways associated with carcinogenesis or cancer progression (Table 3). It is notable that these changes may have been occurring in tissue that was histologically normal, consistent with a “field effect” in the prostate gland, and with studies of whole-genome sequencing of noncancerous prostate tissue (27). The amount of broccoli or glucoraphanin consumed by men in this control arm was below the threshold that has been reported in epidemiological studies to reduce the risk of cancer progression (3), and, likewise, there is no reason to suggest that the low amount of glucoraphanin would have induced these changes.

The changes in gene expression observed in the control dietary arm were suppressed by the diets with soups with enhanced glucoraphanin, in a dose-dependent manner. Thus, consuming soup Y (*Myb28* B/V), delivering intermediate amounts of glucoraphanin, resulted in fewer changes in gene expression, whereas consuming soup Z (*Myb28* V/V), delivering the highest amounts of glucoraphanin, entirely suppressed changes in gene expression seen in the control arm (Table 2, Figure 3). Moreover, GSEA functional pathway analyses of Soup Z were markedly different to those of Soup X (Table 3).

Meta-analyses of epidemiological studies have associated the *GSTM1* null genotype with enhanced risk of prostate cancer (28, 29) and cancer at other sites (30–32), and several epidemiological studies have reported that the beneficial effect of diets rich in cruciferous vegetables in reducing cancer risk is modified by *GSTM1* genotype (33–37). Experimental human dietary intake studies with biological markers have reported greater effect of isothiocyanate intake with *GSTM1* null individuals than those with 1 or 2 *GSTM1* alleles (38, 39). We undertook exploratory analyses of the possible interaction between *GSTM1* genotype and diet by stratifying each arm by genotype and analyzed the changes in paired gene expression and GSEA. We found that changes in gene expression only occurred in *GSTM1* non null individuals. One explanation is that, as with previous studies (38, 39), *GSTM1* null individuals had a greater response to sulforaphane and thus even with the low-dose glucoraphanin diet there was some attenuation of changes in gene expression. However, after GSEA, enrichment scores of pathways were similar in both *GSTM1* genotypes on the control diet (Soup X; Supplemental Figure 6), indicating that if there was any effect of glucoraphanin on gene expression it was insufficient to attenuate oncogenic pathways. Similar results were found after consumption of soups Y and Z, albeit with some indication of an increasing divergence of enrichment of pathways between *GSTM1* genotypes with increasing glucoraphanin content of diet, indicative of a possible diet × gene interaction.

Based upon results from cell and animal model systems we expected an intervention with glucoraphanin (and hence sulforaphane) would induce gene expression in a manner that would reduce the risk of cancer incidence or progression. In contrast, we observed a suppression of changes in gene expression. This finding was only apparent owing to our innovative experimental design, and would not have been evident if we had just compared individuals at a single time point (e.g., 12 mo). There are few reports of sequential global gene expression in model systems. One example is the suppression of changes in gene expression (“transcriptional drift”) by the antidepressant miaserin in *Caenorhabditis elegans* (40, 41), in a somewhat analogous manner to the attenuation of changes in gene expression that was observed with the high-glucoraphanin soups. Moreover, miaserin attenuated an oxidative transcriptional signature (41), and this modulation of redox status was considered to be associated with the reduced transcriptional drift associated with ageing of *C. elegans*. This is analogous to the significant reduction in the enrichment score of the reactive oxygen species pathway induced by the high-glucoraphanin soup (Table 3). It is well established that sulforaphane derived from glucoraphanin induces acute oxidative stress followed by induction of NRF2-regulated genes that modulate cellular redox status (8). In our study, we did not observe any changes in expression of NRF2-regulated genes (Supplemental Table 6). This may be due to the transient nature of the changes in the expression of these genes, with changes only occurring in the few hours directly after consuming the soup. However, the regular (once-weekly) exposure to sulforaphane in the high-glucoraphanin intervention arm may result in the maintenance or improvement of redox status of the prostate tissue that inhibits the changes in gene expression associated with oncogenic pathways that were observed in the control arm.

These data suggest that the putative chemopreventive effects of a diet rich in cruciferous vegetables and glucoraphanin are not mediated by direct effects upon cancerous clones, but through a more generic “antiaging” effect. This would be consistent with the beneficial effect of a diet rich in cruciferous vegetables on other chronic age-related diseases (42, 43). Alternatively, the effects of our intervention could be through epigenetic regulation (44). Broccoli sprouts and sulforaphane have been shown to reduce prostate cancer incidence through reduction of histone deacetylation 3 (HDAC3) in mice, and altering global DNA methylation in prostate cell models (45, 46). Despite these speculations, there is clearly a “mechanistic gap” between the phenomena observed in model systems, that often involve short-term high-dose exposures, and those observed in human studies for which there are several ethical and clinical constraints in study design. This may be partially resolved through improved experimental design in model systems that use longer and lower-dose interventions and sequential analyses of tissues, and more innovative human studies involving analyses of biopsy tissues after precisely timed dietary interventions.

One of the limitations of our trial was the relatively small sample size, resulting from the low accrual rate of eligible patients, and we did not meet our target recruitment to achieve the original power estimation. Having fewer patients in each arm may have decreased the number of genes identified as being differentially expressed at a fixed FDR-adjusted *P* value and moderated the GSEA, but each arm would have been affected equally and it is not likely that under-recruitment could explain the differences between groups that we observed. Obtaining and analyzing sequential paired prostate biopsy samples from the patients rendered our data less susceptible to interindividual variability, thus partly compensating for the reduced sample size. Another limitation was that the biopsies analyzed were all considered nonneoplastic, based on directly adjacent histology. Although this assumption may be erroneous for some of the biopsies, the global transcriptional profiles of all the biopsies within our cohort were more similar to the profiles of primary prostate cancer, suggesting that the whole prostate undergoes transcriptional changes at the onset of prostate cancer.

In conclusion, our data are entirely consistent with epidemiological studies that inversely correlate diets rich in either cruciferous vegetables or glucosinolates with prostate cancer incidence or progression. We report that an intervention rich in glucoraphanin attenuated the transcriptional changes occurring in prostate of men on active surveillance over a period of 12 mo. Although our study was not designed or sufficiently powered to quantify clinical endpoints, we also observed a negative correlation between the intake of cruciferous vegetables and their associated sulfur-metabolites, and the change in WHO grade over time (Figure 4). Further studies are warranted to explore this association, with sufficient volunteer numbers and appropriate follow-up time to assess clinical endpoints in an active surveillance cohort. The results of the study would support a public health recommendation to include cruciferous vegetables as part of the diet to maintain and promote health.

## Supplementary Material

nqz012_Supplemental_Files

## Notes

Supported by Prostate Cancer Foundation Challenge Award, 2012 (to RFM) and UK Biotechnology and Biological Sciences Research Council grant BB/J004545/1 (to RFM).

The funders played no role in the design and performance of the study; collection, management, analysis, and interpretation of data; or preparation, review, and approval of the manuscript.

Supplemental Tables 1–6 and Supplemental Figures 1–6 are available from the “Supplementary data” link in the online posting of the article and from the same link in the online table of contents at https://academic.oup.com/ajcn/.

Abbreviations used: DGE, differential gene expression; ESCAPE, Effect of Sulforaphane on prostate CAncer PrEvention; FDR, false discovery rate; GSEA, gene set enrichment analysis; *GSTM1*, Glutathione S-transferase M1; MDS, multidimensional scaling; MS, mass spectroscopy; NRF2, nuclear factor (erythroid-derived 2)-like 2; PSA, prostate specific antigen; RRHO, rank–rank hypergeometric overlap; SMCSO, S-methyl cysteine sulfoxide; T0, beginning of the intervention; T12, after 12 mo of intervention; TTB, transperineal template biopsy.
