# Supplementary material for: Transcriptional changes in prostate of men on active surveillance after a 12-mo glucoraphanin-rich broccoli intervention—results from the Effect of Sulforaphane on prostate CAncer PrEvention (ESCAPE) randomized controlled trial
Source: Am J Clin Nutr. 2019 Apr 15;109(4):1133–44. doi: 10.1093/ajcn/nqz012 (PMC6462431; doi:10.1093/ajcn/nqz012)
Supplement: nqz012_Supplemental_Files [file nqz012_supplemental_files.zip › AJCN_Online Supporting Material_Figs_draft5.pptx]

## Slide 1
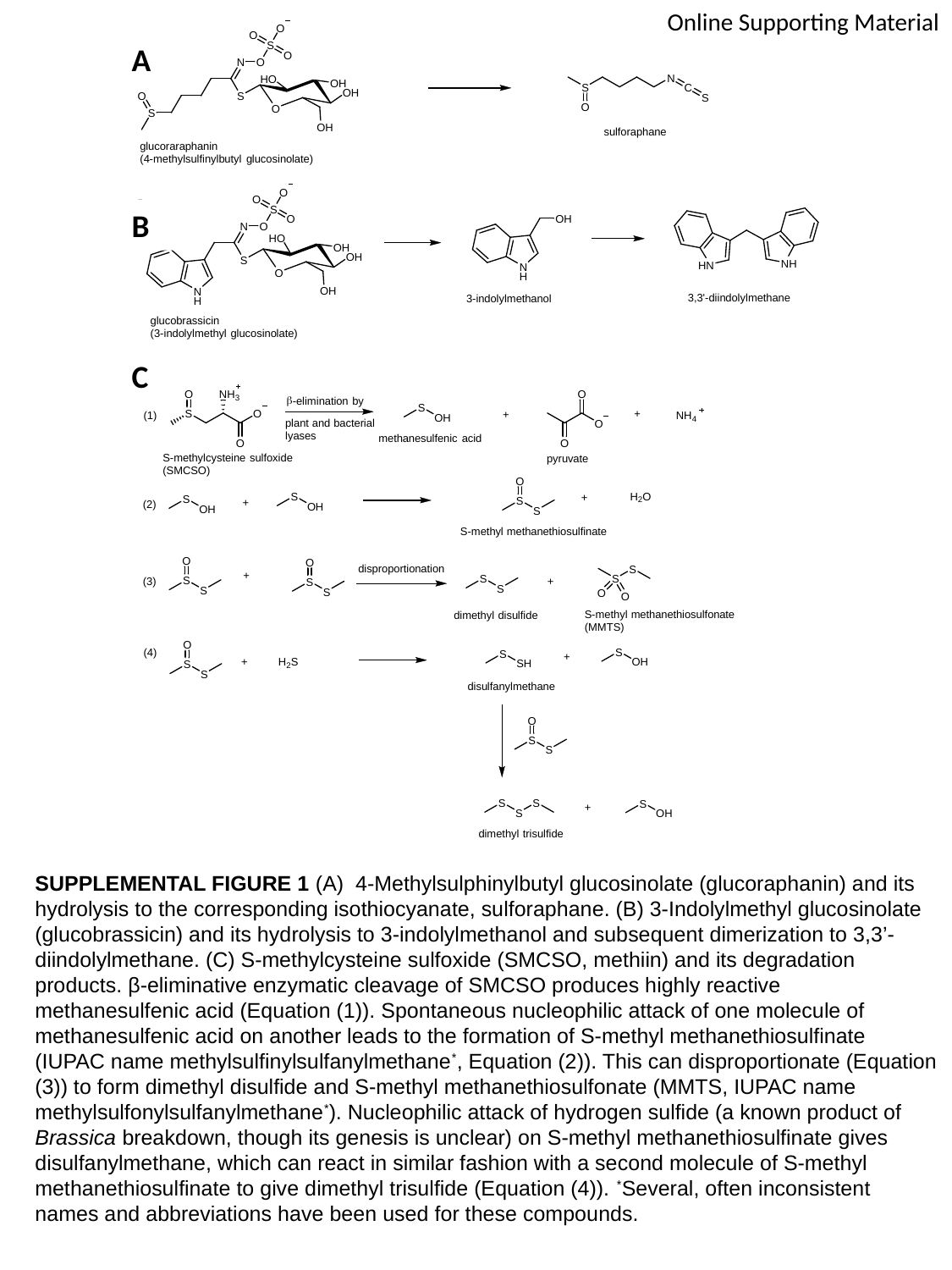

Online Supporting Material
A
B
C
SUPPLEMENTAL FIGURE 1 (A) 4-Methylsulphinylbutyl glucosinolate (glucoraphanin) and its hydrolysis to the corresponding isothiocyanate, sulforaphane. (B) 3-Indolylmethyl glucosinolate (glucobrassicin) and its hydrolysis to 3-indolylmethanol and subsequent dimerization to 3,3’-diindolylmethane. (C) S-methylcysteine sulfoxide (SMCSO, methiin) and its degradation products. β-eliminative enzymatic cleavage of SMCSO produces highly reactive methanesulfenic acid (Equation (1)). Spontaneous nucleophilic attack of one molecule of methanesulfenic acid on another leads to the formation of S-methyl methanethiosulfinate (IUPAC name methylsulfinylsulfanylmethane*, Equation (2)). This can disproportionate (Equation (3)) to form dimethyl disulfide and S-methyl methanethiosulfonate (MMTS, IUPAC name methylsulfonylsulfanylmethane*). Nucleophilic attack of hydrogen sulfide (a known product of Brassica breakdown, though its genesis is unclear) on S-methyl methanethiosulfinate gives disulfanylmethane, which can react in similar fashion with a second molecule of S-methyl methanethiosulfinate to give dimethyl trisulfide (Equation (4)). *Several, often inconsistent names and abbreviations have been used for these compounds.

## Slide 2
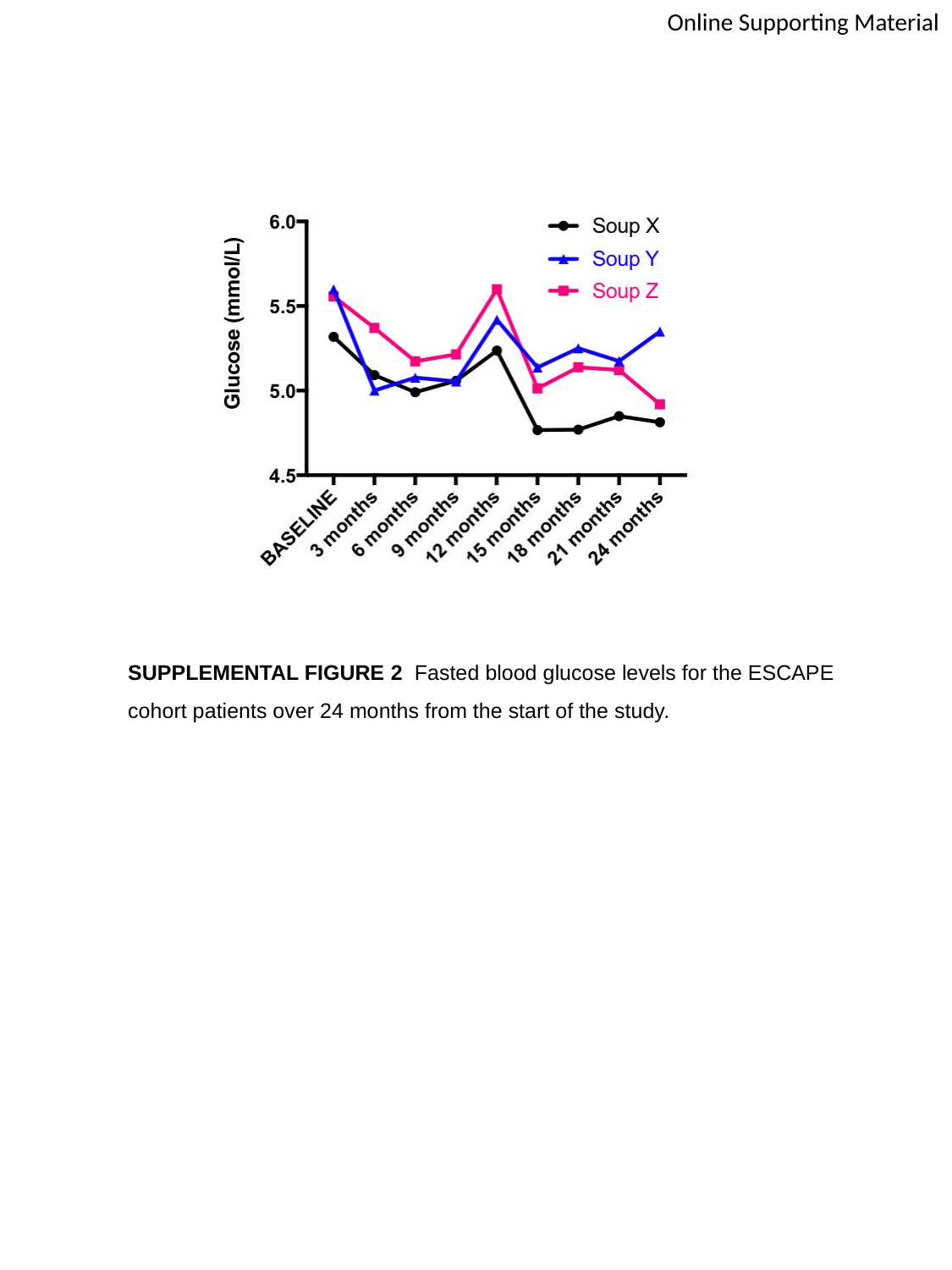

Online Supporting Material
SUPPLEMENTAL FIGURE 2 Fasted blood glucose levels for the ESCAPE cohort patients over 24 months from the start of the study.

## Slide 3
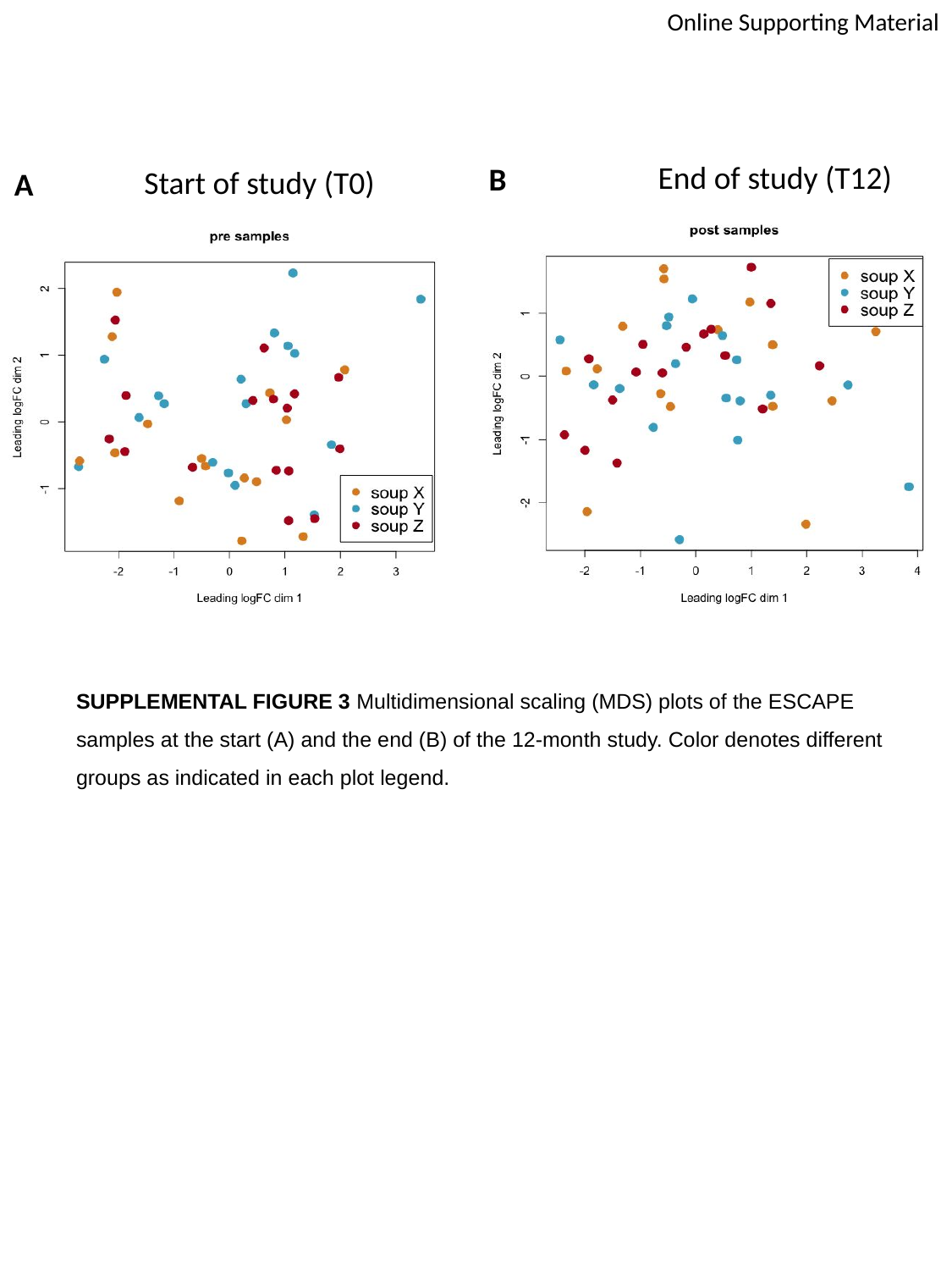

Online Supporting Material
End of study (T12)
B
Start of study (T0)
A
SUPPLEMENTAL FIGURE 3 Multidimensional scaling (MDS) plots of the ESCAPE samples at the start (A) and the end (B) of the 12-month study. Color denotes different groups as indicated in each plot legend.

## Slide 4
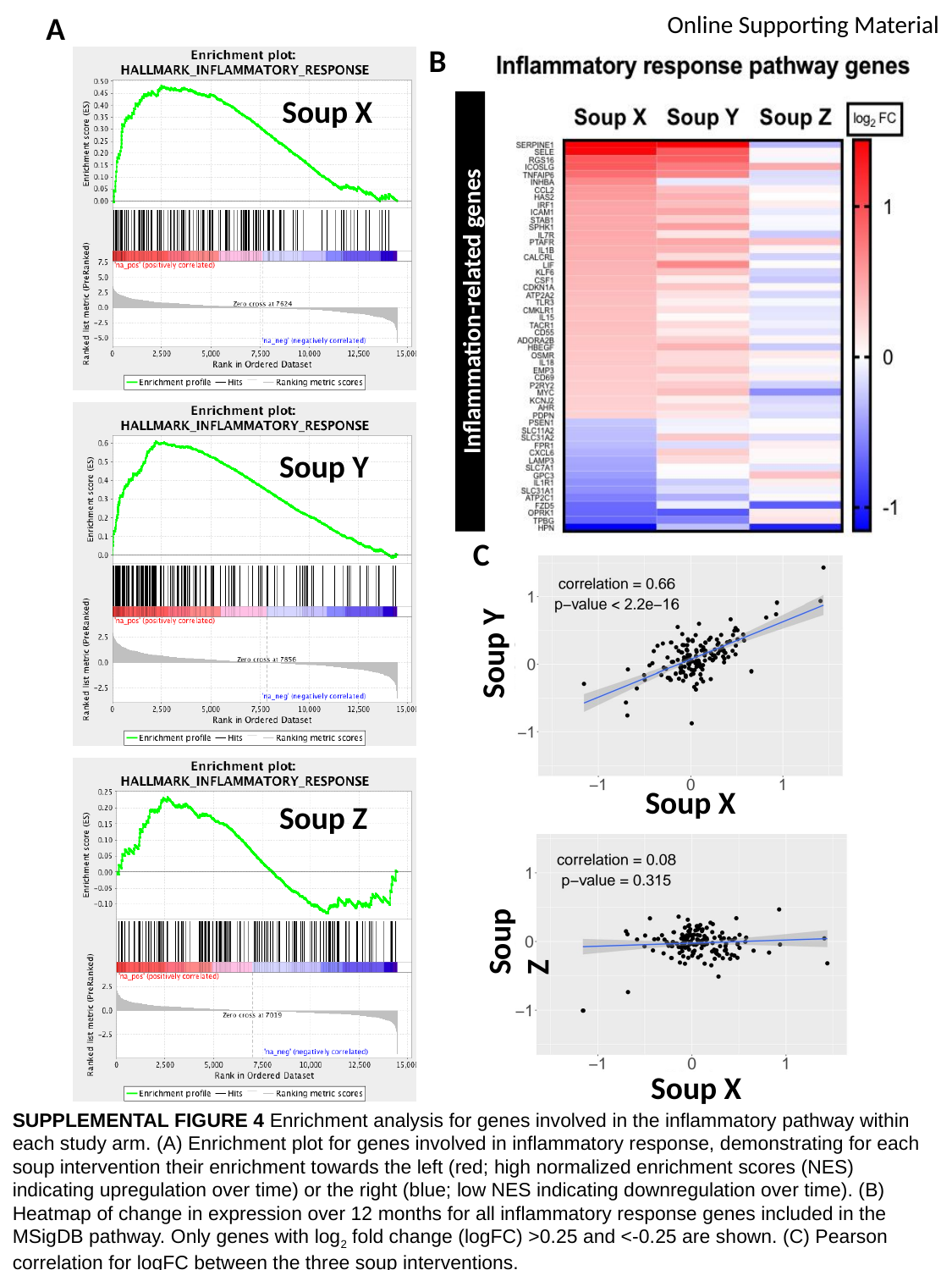

A
Online Supporting Material
B
Soup X
Inflammation-related genes
Soup Y
C
Soup Y
Soup X
Soup Z
Soup Z
Soup X
SUPPLEMENTAL FIGURE 4 Enrichment analysis for genes involved in the inflammatory pathway within each study arm. (A) Enrichment plot for genes involved in inflammatory response, demonstrating for each soup intervention their enrichment towards the left (red; high normalized enrichment scores (NES) indicating upregulation over time) or the right (blue; low NES indicating downregulation over time). (B) Heatmap of change in expression over 12 months for all inflammatory response genes included in the MSigDB pathway. Only genes with log2 fold change (logFC) >0.25 and <-0.25 are shown. (C) Pearson correlation for logFC between the three soup interventions.

## Slide 5
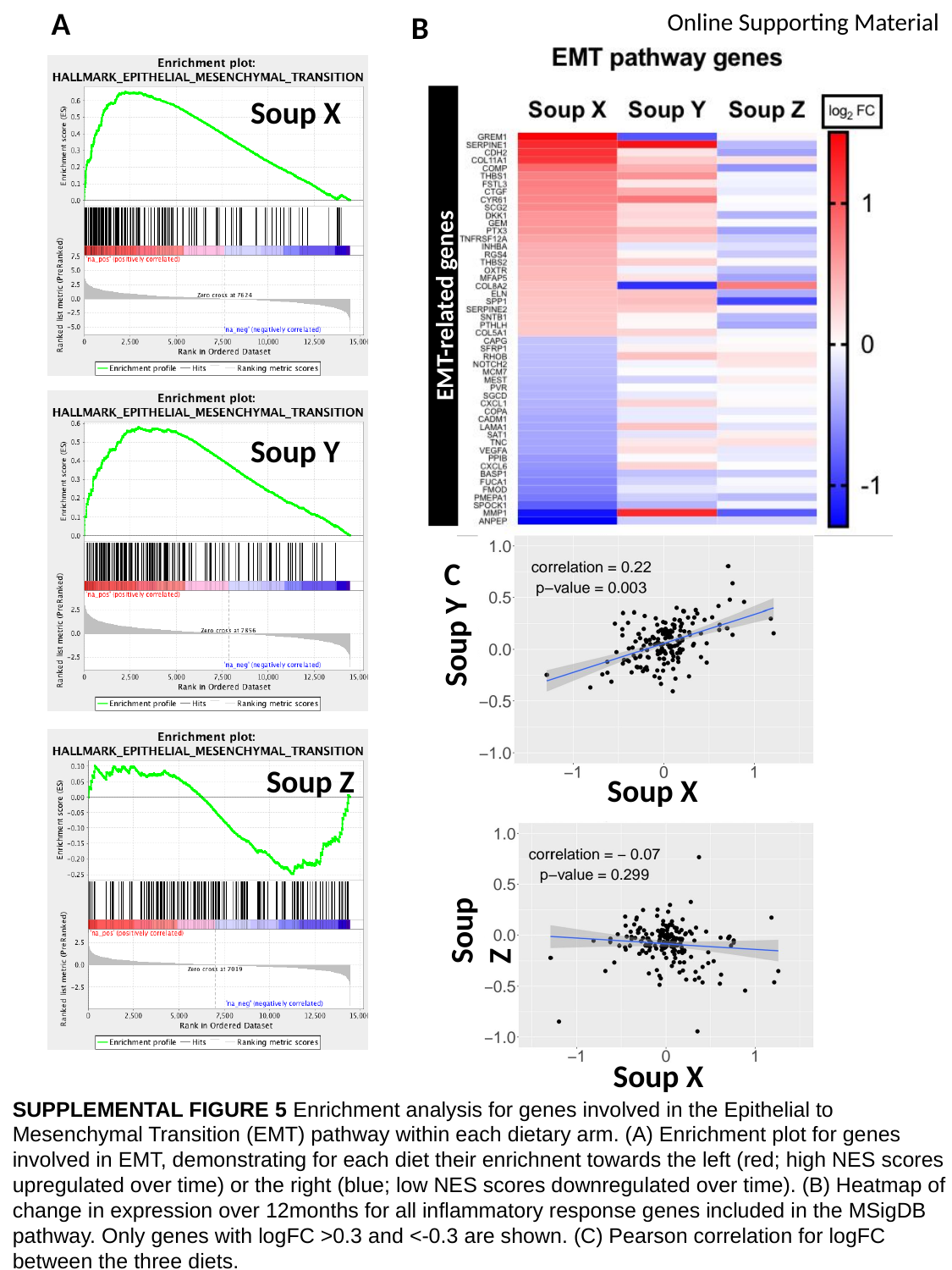

Online Supporting Material
A
B
Soup X
EMT-related genes
Soup Y
C
Soup Y
Soup Z
Soup X
Soup Z
Soup X
SUPPLEMENTAL FIGURE 5 Enrichment analysis for genes involved in the Epithelial to Mesenchymal Transition (EMT) pathway within each dietary arm. (A) Enrichment plot for genes involved in EMT, demonstrating for each diet their enrichnent towards the left (red; high NES scores upregulated over time) or the right (blue; low NES scores downregulated over time). (B) Heatmap of change in expression over 12months for all inflammatory response genes included in the MSigDB pathway. Only genes with logFC >0.3 and <-0.3 are shown. (C) Pearson correlation for logFC between the three diets.

## Slide 6
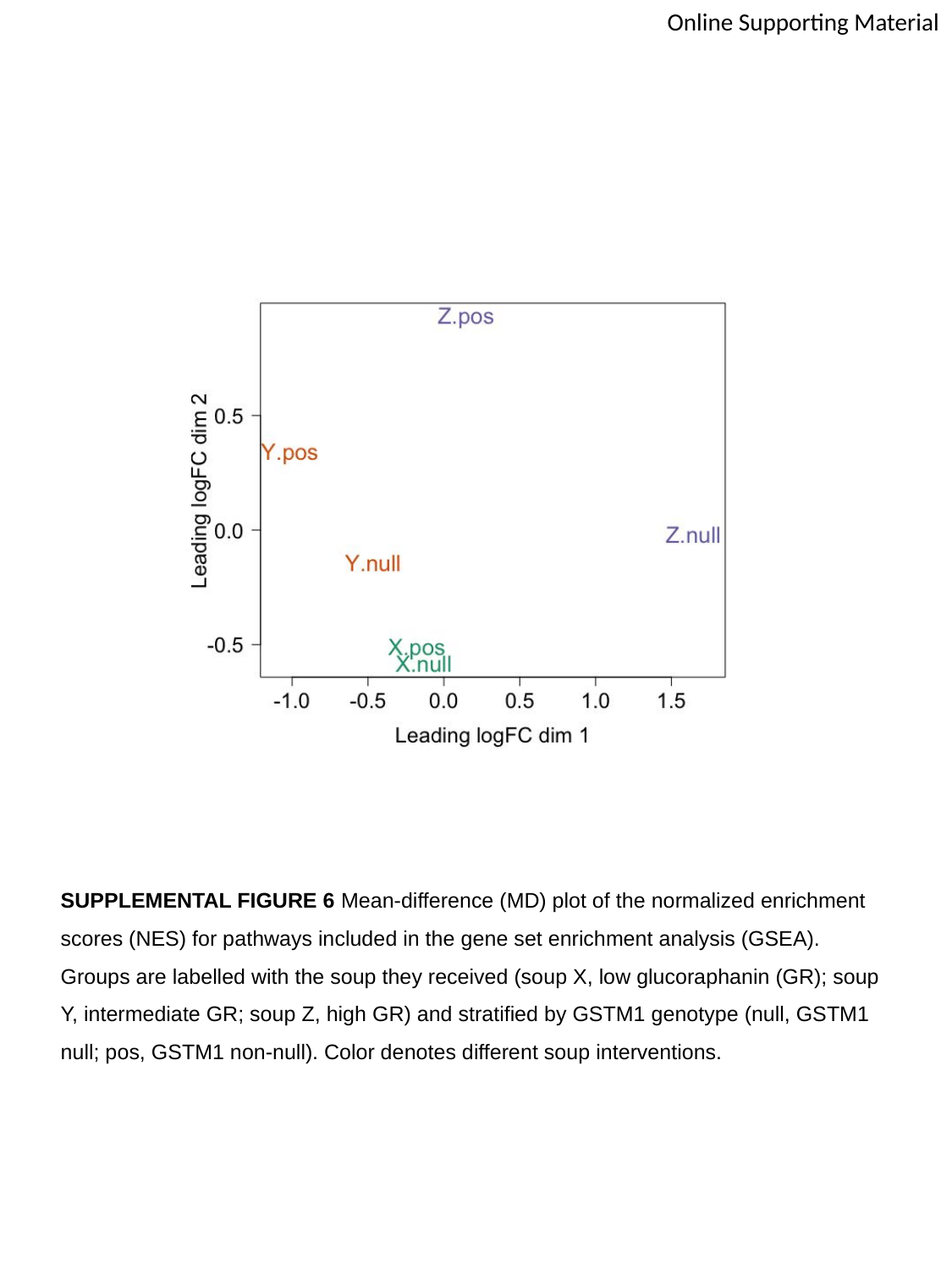

Online Supporting Material
SUPPLEMENTAL FIGURE 6 Mean-difference (MD) plot of the normalized enrichment scores (NES) for pathways included in the gene set enrichment analysis (GSEA). Groups are labelled with the soup they received (soup X, low glucoraphanin (GR); soup Y, intermediate GR; soup Z, high GR) and stratified by GSTM1 genotype (null, GSTM1 null; pos, GSTM1 non-null). Color denotes different soup interventions.
