# Supplementary material for: Transcriptional changes in prostate of men on active surveillance after a 12-mo glucoraphanin-rich broccoli intervention—results from the Effect of Sulforaphane on prostate CAncer PrEvention (ESCAPE) randomized controlled trial
Source: Am J Clin Nutr. 2019 Apr 15;109(4):1133–44. doi: 10.1093/ajcn/nqz012 (PMC6462431; doi:10.1093/ajcn/nqz012)
Supplement: nqz012_Supplemental_Files [file nqz012_supplemental_files.zip › AJCN_Online Supporting Material_Tables_draft4.docx]

**SUPPLEMENTAL TABLE 1**

| **Inclusion and exclusion criteria for the ESCAPE study** |
| --- |
| **Inclusion criteria** |
| 1. Males |
| 1. On active surveillance for low- and intermediate-risk prostate cancer (Gleason score ≤7, PSA ≤20µg/l, stage ≤ T2) |
| 1. Aged 18-80 years |
| 1. BMI between 19.5 and 35 kg/m^2^ |
| 1. Smokers and non-smokers |
| **Exclusion criteria** |
| 1. Those taking 5α-reductase inhibitors or testosterone replacement supplements |
| 1. Those on warfarin treatment |
| 1. Those diagnosed with diabetes |
| 1. Those diagnosed with or suspected to be at high-risk for human immunodeficiency virus (HIV) and/or hepatitis virus infection |
| 1. Those allergic to any of the ingredients of the broccoli soups |
| 1. Those taking dietary supplements or herbal remedies which may affect the trial outcome |
| 1. Parallel participation in another research project that involves dietary intervention |
| 1. Anybody related to or living with any member of the trial team |

**SUPPLEMENTAL TABLE 2**

| Blood parameters for the ESCAPE cohort patients | | | |
| --- | --- | --- | --- |
|  | **Soup X**  **(control; lowGR)** | **Soup Y**  **(intermediate GR)** | **Soup Z**  **(high GR)** |
| N | 15 | 17 | 16 |
| Glucose mmol/L |  |  |  |
| 0 month | 5.2 (5.0 – 5.7) | 5.5 (5.1 – 5.9) | 5.6 (5.2 – 6.0) |
| 12 month | 5.4 (4.8 – 5.5) | 5.6 (5.3 – 6.1) | 5.5 (5.0 – 5.8) |
| Cholesterol mmol/L |  |  |  |
| 0 month | 5.6 (4.9 – 6.5) | 5.2 (4.9 – 5.7) | 5.1 (4.0 – 5.6) |
| 12 month | 5.4 (4.1 – 5.8) | 5.4 (5.0 – 6.2)^a^ | 4.7 (4.2 – 5.6)^a^ |
| HDLC mmol/L |  |  |  |
| 0 month | 1.38 (1.18 – 1.75) | 1.25 (1.15 – 1.43) | 1.24 (1.12 – 1.59) |
| 12 month | 1.36 (1.07 – 1.66) | 1.27 (1.11 – 1.55) | 1.26 (1.03 – 1.63) |
| LDLC mmol/L |  |  |  |
| 0 month | 3.8 (3.0 – 4.1) | 3.4 (3.1 – 3.7) | 3.3 (2.3 – 3.9) |
| 12 month | 3.6 (3.2 – 4.0) | 3.4 (3.0 – 4.0) | 3.4 (2.4 – 3.5) |
| Cholesterol/HDLC |  |  |  |
| 0 month | 4.2 (3.3 – 4.8) | 4.1 (3.7 – 5.5) | 3.5 (3.1 – 4.9) |
| 12 month | 4.0 (3.4 – 4.9) | 4.2 (3.5 – 5.4) | 3.8 (3.2 – 4.7) |
| Triglycerides mmol/L |  |  |  |
| 0 month | 0.86 (0.8 – 1.03) | 1.02 (0.91 – 1.75) | 0.82 (0.65 – 1.33) |
| 12 month | 0.63 (0.77 – 1.16)^a^ | 1.32 (1.01 – 1.69)^a^ | 0.95 (0.72 – 1.52) |
| Albumin g/L |  |  |  |
| 0 month | 39 (38 – 41) | 41 (39 – 42) | 39 (37 – 42) |
| 12 month | 39 (37 – 41) | 41 (39 – 42) | 39 (37 – 42) |
| ALP U/L |  |  |  |
| 0 month | 69.5 (52 – 75) | 62 (54 – 75) | 69 (62.5 – 81) |
| 12 month | 73.5 (56 – 82) | 66.5 (59.5 – 80) | 70 (63 – 78) |
| ALT U/L |  |  |  |
| 0 month | 15.5 (14 – 23) | 19 (15 – 27) | 31 (30 – 33) |
| 12 month | 15.5 (15 – 25)^a^ | 21 (15.5 – 25.5) | 31 (28 – 33)^a^ |
| Globulin g/L |  |  |  |
| 0 month | 31 (29 – 32) | 29 (28 – 31) | 31 (30 – 33) |
| 12 month | 30.5 (28 – 32) | 29 (28 – 32) | 31 (28 – 33) |
| Bilirubin µmol/L |  |  |  |
| 0 month | 13.5 (9.5 – 17) | 14 (10 – 18) | 10.5 (9 – 16) |
| 12 month | 12 (9 – 17.5) | 13.5 (9.5 – 19) | 12 (9 – 15) |
| Protein g/L |  |  |  |
| 0 month | 70 (68 – 71) | 70 (67 – 72) | 71 (68 – 73) |
| 12 month | 68.5 (66 – 73) | 70 (67 – 73) | 69.5 (67 – 72.5) |
| Na mmol/L |  |  |  |
| 0 month | 141 (140 – 142) | 141 (139 – 142) | 141 (139 – 141) |
| 12 month | 141 (140 – 142) | 140 (139 – 143) | 140 (138 – 141) |
| K mmol/L |  |  |  |
| 0 month | 4.2 (4.0 – 4.4) | 4.1 (3.9 – 4.4)^a^ | 4.5 (4.2 – 4.8)^a^ |
| 12 month | 4.4 (4.3 – 4.5) | 4.3 (4.1 – 4.5) | 4.4 (4.0 – 4.6) |
| Urea mmol/L |  |  |  |
| 0 month | 5.8 (4.1 – 7.3) | 5.5 (4.4 – 6.3) | 5.8 (4.8 – 6.5) |
| 12 month | 5.2 (4.4 – 6.5) | 5.8 (5.6 – 6.6) | 5.1 (4.8 – 6.0) |
| Creatinine µmol/L |  |  |  |
| 0 month | 82 (77 – 88) | 91 (84 – 95) | 90 (74 – 101) |
| 12 month | 80 (76 – 85)^a^ | 92 (74 – 92)^a^ | 90 (73 – 95) |
| eGFR mUmin/1.73m^2^ |  |  |  |
| 0 month | 80 (75 – 87) | 73 (69 – 82) | 75 (74 – 101) |
| 12 month | 83 (77 – 86) | 73 (69 – 80) | 72 (69 – 90) |
| Wbc 10^9^/L |  |  |  |
| 0 month | 5.6 (5.3 – 6.5) | 5.2 (4.7 – 5.9) | 5.9 (4.9 – 6.5) |
| 12 month | 5.7 (5.3 – 6.9) | 5.8 (4.8 – 6.2) | 6.4 (5.5 – 7.5) |
| Rbc 10^12^/L |  |  |  |
| 0 month | 5.04 (4.62 – 5.15) | 4.95 (4.70 – 5.16) | 4.98 (4.78 – 5.33) |
| 12 month | 5.03 (4.92 – 5.31) | 4.95 (4.91 – 5.28) | 5.17 (5.00 – 5.32) |
| Hb g/L |  |  |  |
| 0 month | 153 (146 – 155) | 150 (147 – 155) | 150 (147 – 158) |
| 12 month | 151 (145 – 156) | 151 (146 – 150) | 155 (144 – 160) |
| Hct % |  |  |  |
| 0 month | 43.3 (42.8 – 46.1) | 43.7 (41.9 – 45.5) | 43.4 (42.4 – 47.1) |
| 12 month | 44.7 (43.9 – 45.4) | 44.9 (42.8 – 46.2) | 43.9 (43.1 – 46.9) |
| MCV fL |  |  |  |
| 0 month | 89 (86 – 91) | 89 (82 – 91) | 88 (85 – 91) |
| 12 month | 90 (87 – 91) | 88 (87 – 92) | 87 (84 – 91) |
| MCH pg |  |  |  |
| 0 month | 30.1 (29.6 – 31.4) | 31.0 (29.6 – 31.7) | 30.2 (29.5 – 31.6) |
| 12 month | 30.1 (29.4 – 31.1) | 30.6 (28.9 – 32.2) | 30.4 (28.4 – 31.0) |
| Platelets 10^9^/L |  |  |  |
| 0 month | 214 (184 – 262) | 216 (179 – 273) | 212 (162 – 277) |
| 12 month | 210 (180 – 247) | 218 (181 – 293) | 215 (146 – 251) |
| Neutrophils 10^9^/L |  |  |  |
| 0 month | 2.87 (2.59 – 3.69) | 3.09 (2.48 – 3.33) | 3.67 (2.86 – 3.96) |
| 12 month | 3.15 (2.52 – 3.87) | 3.01 (2.38 – 3.55) | 3.90 (3.34 – 4.54) |
| Neutrophils 10^9^/L |  |  |  |
| 0 month | 2.87 (2.59 – 3.69) | 3.09 (2.48 – 3.33) | 3.67 (2.86 – 3.96) |
| 12 month | 3.15 (2.52 – 3.87) | 3.01 (2.38 – 3.55) | 3.90 (3.34 – 4.54) |
| Lymphocytes 10^9^/L |  |  |  |
| 0 month | 1.61 (1.45 – 1.96) | 1.61 (1.28 – 1.81) | 1.55 (1.21 – 1.80) |
| 12 month | 1.58 (1.44 – 2.23) | 1.66 (1.32 – 2.01) | 1.84 (1.28 – 1.99) |
| Monocytes 10^9^/L |  |  |  |
| 0 month | 0.52 (0.45 – 0.60) | 0.47 (0.37 – 0.52)^a^ | 0.55 (0.52 – 0.74)^a^ |
| 12 month | 0.52 (0.46– 0.67) | 0.51 (0.39 – 0.58) | 0.58 (0.50 – 0.72) |
| Eosinophils 10^9^/L |  |  |  |
| 0 month | 0.27 (0.12 – 0.44) | 0.16 (0.13 – 0.20) | 0.15 (0.11 – 0.23) |
| 12 month | 0.23 (0.14 – 0.37)^a^ | 0.16 (0.12 – 0.34) | 0.14 (0.10 – 0.27)^a^ |
| Basophils 10^9^/L |  |  |  |
| 0 month | 0.05 (0.03 – 0.07) | 0.04 (0.05 – 0.06) | 0.05 (0.02 – 0.06) |
| 12 month | 0.06 (0.04 – 0.07) | 0.05 (0.03 – 0.06) | 0.04 (0.03 – 0.06) |
| Data shown are median (IQR). Within rows, all values are not significantly different (P values >0.05, Kruskal Wallis), except pairs followed by ^a^ in which 0.01> *P* >0.05 (Kruskal Wallis and Dunn’s multiple comparisons) | | | |

**SUPPLEMENTAL TABLE 3**

| Weekly intake levels of dietary components for the ESCAPE cohort patients | | | | | | | |  |
| --- | --- | --- | --- | --- | --- | --- | --- | --- |
| Dietary component | Time of assessment | Soup X |  | Soup Y |  | Soup Z | *P*-value^a^ | |
| Protein (g) | *baseline* | 555 ± 135 |  | 571 ± 145 |  | 613 ± 202 | ns | |
|  | *study* | 545 ± 101 |  | 591 ± 166 |  | 632 ± 130 | ns | |
| fat (g) | *baseline* | 549 ± 186 |  | 553 ± 204 |  | 625 ± 315 | ns | |
|  | *study* | 539 ± 137 |  | 552 ± 154 |  | 658 ± 203 | ns | |
| Carbohydrate (g) | *baseline* | 1589 ±407 |  | 1774 ± 454 |  | 1766 ± 688 | ns | |
|  | *study* | 1519 ± 432 |  | 1623 ± 423 |  | 1903 ± 485 | ns | |
| energy (GJ) | *baseline* | 58.2 ±15.0 |  | 62.3 ± 15.9 |  | 65.2 ± 24.9 | ns | |
|  | *study* | 56.4 ± 12.5 |  | 59.8 ± 13.4 |  | 68.1 ± 15.5 | ns | |
| Total sugars (g) | *baseline* | 661 ± 259 |  | 760 ± 254 |  | 829 ±429 | ns | |
|  | *study* | 647 ± 256 |  | 690 ± 231 |  | 843 ± 373 | ns | |
| Alcohol (g) | *baseline* | 104 ±88 |  | 127 ± 72 |  | 81 ± 102 | ns | |
|  | *study* | 100 ± 81 |  | 156 ± 181 |  | 88 ± 86 | ns | |
| Dietary fibre total AOAC | *baseline* | 158 ± 43 |  | 163 ± 56 |  | 163 ±85 | ns | |
|  | *study* | 138 ± 41 |  | 158 ± 47 |  | 176 ± 62 | ns | |
| Calcium (g) | *baseline* | 6.2 ± 2.0 |  | 7.1 ± 2.7 |  | 7.4 ± 3.3 | ns | |
|  | *study* | 6.3 ± 1.9 |  | 6.5 ± 2.6 |  | 7.4 ± 2.6 | ns | |
| Zinc (mg) | *baseline* | 67 ± 20 |  | 70 ± 19 |  | 70 ±25 | ns | |
|  | *study* | 62 ± 14 |  | 73 ± 24 |  | 74 ± 17 | ns | |
| Selenium (µg) | *baseline* | 368 ± 135 |  | 362 ± 129 |  | 448 ± 228 | ns | |
|  | *study* | 358 ± 111 |  | 350 ± 92 |  | 423 ± 102 | ns | |
| Carotene (mg) | *baseline* | 28 ± 18 |  | 31 ± 20 |  | 32 ± 19 | ns | |
|  | *study* | 21 ± 10 |  | 27 ± 14 |  | 30 ± 17 | ns | |
| Vitamin D (µg) | *baseline* | 22 ± 10 |  | 27 ± 18 |  | 16 ± 7 | ns | |
|  | *study* | 21 ± 9 |  | 21 ± 12 |  | 21 ± 8 | ns | |
| Vitamin E (mg) | *baseline* | 72 ±37 |  | 78 ± 31 |  | 77 ± 48 | ns | |
|  | *study* | 60 ± 29 |  | 72 ± 30 |  | 78 ± 29 | ns | |
| Tomato (g) | *baseline* | 283 ± 290 |  | 345 ± 262 |  | 283 ±496 | ns | |
|  | *study* | 230 ± 253 |  | 256 ± 225 |  | 335 ± 344 | ns | |
| White fish (g) | *baseline* | 117 ± 105 |  | 96 ± 89 |  | 139 ±137 | ns | |
|  | *study* | 154 ± 94 |  | 82 ± 94 |  | 124 ± 96 | ns | |
| Oily fish (g) | *baseline* | 116 ± 110 |  | 95 ± 146 |  | 85 ±107 | ns | |
|  | *study* | 112 ± 115 |  | 88 ± 123 |  | 82 ± 103 | ns | |
| Cruciferous vegtables (g) | *baseline* | 290 ± 186 |  | 328 ± 277 |  | 188 ± 173 ^b^ | ns | |
|  | *study* | 261 ± 149 |  | 343 ± 156 |  | 280 ± 117 ^b^ | ns | |
| Alliiacous vegetables (g) | *baseline* | 81 ± 137 |  | 82 ± 89 |  | 59 ±102 | ns | |
|  | *study* | 56 ± 40 |  | 70 ± 42 |  | 93 ± 112 | ns | |
| ITC-glucosinolates (µmol) | *baseline* | 269 ± 170 |  | 312 ± 200 ^c^ |  | 164 ± 149 ^d^ | ns | |
|  | *study* | 274 ± 193 |  | 490 ± 184 ^c^ |  | 675 ± 136 ^d^ | <0.0001 | |
| Glucoraphanin delivered by  the intervention soups (µmol) | *baseline* | - |  | - |  | - |  | |
|  | *study* | 72 ± 2.8 |  | 214 ± 7.3 |  | 492 ± 3.2 | <0.0001 | |
| Indole glucosinolates (µmol) | *baseline* | 82 ±59 |  | 107 ± 141 |  | 97 ± 122 | ns | |
|  | *study* | 94 ± 65 |  | 90 ± 39 |  | 161 ± 66 | 0.0014 | |
| SMCSO (µmol) | *baseline* | 3221 ± 174 |  | 3821 ± 2966 |  | 2092 ±1883 ^e^ | ns | |
|  | *study* | 2966 ± 1451 |  | 4210 ± 2003 |  | 3761 ± 1608 ^e^ | ns | |
| Data are mean ± SD; *baseline* refers to the intake levels of dietary components at the start of the study (0 months) and *study* refers to the average intake levels between 6 months and 12 months.  ^a^ *P*-value from ANOVA test comparing the intake of dietary components between the three diets. Only those < 0.05 are shown.  ^b,c,d,e^ significant paired *P*-values between the start and the end of the study (paired *P*-value < 0.05); ^b^ paired *P*-value = 0.029; ^c^ paired *P*-value = 0.0012; ^d^ paired *P*-value < 0.0001; ^e^ paired *P*-value = 0.012  ITC = isothiocyanates; SMCSO = S-methyl cysteine sulfoxide | | | | | | | |  |

| Number of genes changed over time with the different diets stratified by GSTM1 genotype | | | | | | | |
| --- | --- | --- | --- | --- | --- | --- | --- |
|  |  | Soup X (*n* = 15) | | Soup Y (*n* = 17) | | Soup Z (*n* = 16) | |
|  |  | **GSTM1 null (n=9)** | **GSTM1 non null (n=6)** | **GSTM1 null (n=10)** | **GSTM1 non null (n=7)** | **GSTM1 null (n=12)** | **GSTM1 non null (n=4)** |
| **FDR-adjusted *P*-value^1^** | <0.05 | 0 | 2↓ | 0 | 1↑ | 0 | 0 |
|  | <0.1 | 0 | 104↑, 48↓ | 0 | 4↑, 1↓ | 0 | 1↑, 2↓ |
|  | <0.2 | 0 | 477↑, 302↓ | 0 | 29↑, 27↓ | 0 | 1↑, 2↓ |
|  | <0.5 | 0 | 2828↑, 2440↓ | 0 | 2586↑, 1990↓ | 1↑ | 2↑, 3↓ |
| ***P*-value^2^** | <0.001 | 6↑, 5↓ | 105↑, 53↓ | 3↑, 4↓ | 38↑, 34↓ | 9↑, 5↓ | 4↑, 6↓ |
|  | <0.01 | 36↑, 52↓ | 488↑, 309↓ | 59↑, 55↓ | 330↑, 339↓ | 53↑, 41↓ | 54↑, 46↓ |
|  | <0.05 | 315↑, 382↓ | 1389↑, 1067↓ | 353↑, 338↓ | 1258↑, 1091↓ | 282↑, 246↓ | 330↑, 309↓ |
| ^1^ paired T-tests, adjusted for multiple testing correction by Benjamini-Hochberg (BH)  ^2^ Students paired T-tests, unadjusted for multiple testing correction  GSTM1 = glutathione S-transferase | | | | | | | |

**SUPPLEMENTAL TABLE 4**

**SUPPLEMENTAL TABLE 5**

| Gene Set Enrichment Analysis (GSEA) of paired changes over time for the control (soup X, low GR) and the experimental arms, soup Y (intermediate GR) and soup Z (high GR) stratified by GSTM1 genotype | | | | | | | | | | | | | | |
| --- | --- | --- | --- | --- | --- | --- | --- | --- | --- | --- | --- | --- | --- | --- |
|  |  | Soup X  GSTM1 null | | Soup X  GSTM1 non null | | Soup Y  GSTM1 null | | Soup Y  GSTM1 non null | | Soup Z  GSTM1 null | | | Soup Z  GSTM1 non null | |
| NAME | SIZE | NES | q-value | NES | q-value^1^ | NES | q-value^1^ | NES | q-value^1^ | NES | q-value | NES | | q-value^1^ |
| Myogenesis | 173 | 2.20 | <0.001 | 1.66 | 0.003 | 1.73 | 0.003 | 1.44 | 0.024 | -1.47 | 0.037 | -2.33 | | <0.001 |
| Protein secretion | 91 | -2.12 | <0.001 | -1.88 | 0.005 | -1.45 | 0.095 | -0.82 | 0.893 | -1.60 | 0.015 | -0.96 | | 0.681 |
| Epithelial mesenchymal transition | 176 | 2.54 | <0.001 | 2.52 | <0.001 | 1.62 | 0.008 | 2.23 | <0.001 | -1.11 | 0.315 | 0.78 | | 1 |
| Androgen response | 93 | -2.03 | <0.001 | -1.82 | 0.003 | -2.10 | <0.001 | 1.12 | 0.289 | -1.35 | 0.069 | -0.94 | | 0.667 |
| TNFa signaling via NFKB | 167 | 2.05 | <0.001 | 2.88 | <0.001 | 2.60 | 0 | 2.48 | <0.001 | -1.87 | <0.001 | 1.63 | | 0.009 |
| TGF beta signaling | 46 | 1.94 | <0.001 | 2.00 | <0.001 | 0.88 | 0.836 | 1.56 | 0.008 | -0.96 | 0.572 | 0.97 | | 0.744 |
| Apical junction | 163 | 1.84 | <0.001 | 1.24 | 0.136 | 1.60 | 0.008 | 1.77 | <0.001 | -1.01 | 0.472 | -1.16 | | 0.348 |
| UV response DN | 133 | 1.93 | <0.001 | 2.07 | <0.001 | 1.39 | 0.051 | 1.10 | 0.309 | 1.03 | 1 | -0.94 | | 0.656 |
| Notch signaling | 29 | 1.69 | 0.005 | 1.14 | 0.254 | -1.29 | 0.242 | 1.79 | <0.001 | 0.91 | 1 | 0.68 | | 0.986 |
| Unfolded protein response | 98 | -1.73 | 0.006 | -1.57 | 0.015 | -0.96 | 0.884 | 1.73 | <0.001 | -1.56 | 0.019 | -0.92 | | 0.671 |
| Hypoxia | 164 | 1.61 | 0.010 | 2.02 | <0.001 | 1.68 | 0.004 | 1.70 | 0.002 | -1.02 | 0.471 | -0.97 | | 0.695 |
| Inflammatory response | 152 | 1.60 | 0.010 | 2.11 | <0.001 | 2.40 | 0 | 1.91 | <0.001 | -1.42 | 0.041 | 2.06 | | <0.001 |
| Angiogenesis | 30 | 1.61 | 0.011 | 1.71 | 0.002 | 1.96 | 0 | 1.79 | <0.001 | -1.62 | 0.012 | -1.07 | | 0.446 |
| Coagulation | 89 | 1.62 | 0.011 | 1.91 | <0.001 | 1.46 | 0.027 | 1.73 | <0.001 | -0.93 | 0.639 | 0.92 | | 0.842 |
| Apoptosis | 140 | 1.55 | 0.015 | 1.71 | 0.002 | 1.32 | 0.091 | 1.86 | <0.001 | -1.45 | 0.034 | 1.10 | | 0.513 |
| Wnt beta catenin signaling | 37 | 1.53 | 0.017 | 1.05 | 0.389 | -1.03 | 0.886 | 1.72 | 0.002 | 0.57 | 0.997 | -1.28 | | 0.211 |
| IL2 STAT5 signaling | 154 | 1.48 | 0.026 | 2.02 | <0.001 | 1.79 | 0.001 | 1.78 | <0.001 | -1.51 | 0.029 | 1.11 | | 0.524 |
| Cholesterol homeostasis | 63 | -1.51 | 0.034 | -1.83 | 0.004 | -1.70 | 0.010 | 1.39 | 0.038 | -1.25 | 0.134 | -1.64 | | 0.024 |
| Peroxisome | 82 | -1.53 | 0.034 | -1.72 | 0.004 | -0.95 | 0.849 | 1.07 | 0.347 | -1.02 | 0.460 | -1.10 | | 0.413 |
| Fatty acid metabolism | 127 | -1.48 | 0.035 | -1.75 | 0.004 | -0.91 | 0.930 | 0.91 | 0.680 | -1.31 | 0.092 | -1.45 | | 0.080 |
| G2M checkpoint | 159 | -1.42 | 0.059 | -0.89 | 0.758 | 0.75 | 0.952 | 1.08 | 0.348 | -1.41 | 0.047 | 1.39 | | 0.078 |
| MYC targets v1 | 178 | -1.37 | 0.081 | -1.51 | 0.021 | -1.00 | 0.811 | 1.65 | 0.003 | -2.48 | 0 | 1.04 | | 0.661 |
| KRAS signaling up | 157 | 1.34 | 0.092 | 2.00 | <0.001 | 1.72 | 0.002 | 1.49 | 0.015 | -1.47 | 0.034 | 1.81 | | 0.001 |
| IL6 JAK STAT3 signaling | 61 | 1.33 | 0.093 | 1.67 | 0.003 | 1.92 | <0.001 | 1.99 | <0.001 | -1.47 | 0.033 | 1.90 | | <0.001 |
| UV response UP | 128 | 1.31 | 0.106 | 0.96 | 0.584 | 1.24 | 0.155 | 1.83 | <0.001 | -2.00 | <0.001 | 0.85 | | 0.955 |
| Reactive oxygen species pathway | 42 | -1.30 | 0.118 | -0.90 | 0.786 | 1.05 | 0.411 | 1.38 | 0.041 | -1.64 | 0.011 | -1.18 | | 0.356 |
| MTORC1 signaling | 179 | -1.29 | 0.120 | -1.32 | 0.095 | -1.22 | 0.350 | 1.62 | 0.004 | -1.90 | <0.001 | 1.21 | | 0.271 |
| Mitotic spindle | 171 | 1.19 | 0.252 | 1.51 | 0.015 | 0.94 | 0.693 | 1.55 | 0.008 | 1.38 | 0.250 | -1.13 | | 0.348 |
| P53 pathway | 171 | 1.15 | 0.303 | 1.40 | 0.039 | 1.98 | 0 | 1.86 | 0 | -1.01 | 0.464 | 0.77 | | 1 |
| Interferon gamma response | 162 | 1.10 | 0.404 | 2.19 | 0 | 2.57 | 0 | 1.83 | <0.001 | -1.46 | 0.033 | 1.84 | | 0.001 |
| Estrogen response early | 167 | 1.07 | 0.447 | 1.26 | 0.124 | 1.07 | 0.388 | 1.98 | 0 | -1.11 | 0.324 | 0.85 | | 1 |
| Xenobiotic metabolism | 139 | -1.04 | 0.531 | -1.41 | 0.043 | -0.86 | 1 | 1.71 | 0.002 | -1.49 | 0.031 | -1.14 | | 0.369 |
| Allograft rejection | 146 | 1.02 | 0.561 | 1.85 | <0.001 | 2.30 | 0 | 1.49 | 0.015 | -1.26 | 0.127 | 2.41 | | 0 |
| Complement | 144 | 0.98 | 0.649 | 1.70 | 0.002 | 2.03 | 0 | 1.53 | 0.011 | -1.18 | 0.222 | 1.96 | | <0.001 |
| Estrogen response late | 162 | 0.98 | 0.651 | -1.01 | 0.533 | 1.10 | 0.329 | 1.71 | 0.001 | -1.22 | 0.159 | 1.02 | | 0.653 |
| Glycolysis | 164 | -0.93 | 0.734 | -1.25 | 0.147 | -0.82 | 0.971 | 1.63 | 0.003 | -1.39 | 0.053 | -0.96 | | 0.665 |
| MYC targets v2 | 51 | -0.91 | 0.761 | -1.02 | 0.522 | 0.77 | 0.962 | 2.05 | 0 | -1.77 | 0.002 | -1.17 | | 0.353 |
| E2F targets | 147 | -0.85 | 0.847 | -0.98 | 0.578 | 0.97 | 0.629 | 1.18 | 0.201 | -1.17 | 0.217 | 1.78 | | 0.002 |
| Adipogenesis | 164 | 0.88 | 0.874 | -1.20 | 0.210 | -0.84 | 0.982 | 1.32 | 0.071 | -1.74 | 0.002 | -1.52 | | 0.051 |
| DNA repair | 117 | 0.89 | 0.890 | -1.62 | 0.011 | 0.86 | 0.854 | 1.27 | 0.102 | -1.06 | 0.384 | -1.19 | | 0.370 |
| Interferon alpha response | 81 | -0.74 | 0.959 | 1.73 | 0.002 | 2.18 | 0 | 1.41 | 0.033 | -1.58 | 0.018 | 1.37 | | 0.084 |
| Hedgehog signaling | 30 | 0.82 | 0.987 | 1.20 | 0.169 | 1.30 | 0.100 | 1.46 | 0.021 | 0.83 | 1 | -1.63 | | 0.021 |
| Oxidative phosphorylation | 174 | 0.65 | 1 | -1.42 | 0.041 | -0.77 | 0.979 | 1.00 | 0.485 | -2.18 | 0 | -1.30 | | 0.207 |
| ^1^ Gene Set Enrichment Analysis by GSEA v3.0 on all genes ranked by the significance of fold change (see M&Ms for details). Only pathways significant at *q*-value <0.05 in at least one of the groups are shown.  GR = glucoraphanin; MSigDb = Molecular Signature Database; NES = Normalized Enrichment Score; *q* = FDR-adjusted *P* as described in (22). | | | | | | | | | | | | | | |

**SUPPLEMENTAL TABLE 6**

| Expression of NRF2-associated genes over 12 months in paired samples | | | | | | | | | |  |
| --- | --- | --- | --- | --- | --- | --- | --- | --- | --- | --- |
|  |  | Soup X | |  | Soup Y | |  | Soup Z | | |
| Gene Name | EnsemblID | logFC | *q-*value^a^ |  | logFC | q-value^a^ |  | logFC | q-value^a^ | |
| SRXN1 | ENSG00000271303 | -1.14 | 0.099 |  | 0.08 | 0.880 |  | -0.05 | 0.950 | |
| IDH1 | ENSG00000138413 | -0.52 | 0.099 |  | -0.26 | 0.435 |  | 0.02 | 0.950 | |
| ALDH1A1 | ENSG00000165092 | 0.36 | 0.099 |  | 0.11 | 0.715 |  | 0.08 | 0.950 | |
| NFE2L2 | ENSG00000116044 | 0.20 | 0.099 |  | 0.14 | 0.398 |  | 0.15 | 0.950 | |
| ABCC4 | ENSG00000125257 | -0.83 | 0.099 |  | -0.21 | 0.715 |  | -0.19 | 0.950 | |
| CBR1 | ENSG00000159228 | -0.46 | 0.115 |  | -0.38 | 0.398 |  | -0.25 | 0.950 | |
| PLA2G7 | ENSG00000146070 | -0.67 | 0.115 |  | -0.49 | 0.398 |  | -0.10 | 0.950 | |
| PPARGC1B | ENSG00000155846 | 0.44 | 0.115 |  | 0.10 | 0.742 |  | 0.15 | 0.950 | |
| GLS | ENSG00000115419 | 0.29 | 0.116 |  | 0.09 | 0.715 |  | -0.03 | 0.950 | |
| GLRX | ENSG00000173221 | 0.20 | 0.116 |  | 0.06 | 0.715 |  | -0.05 | 0.950 | |
| GCLC | ENSG00000001084 | -0.22 | 0.116 |  | -0.11 | 0.591 |  | 0.05 | 0.950 | |
| ALDH7A1 | ENSG00000164904 | -0.24 | 0.131 |  | -0.04 | 0.868 |  | -0.03 | 0.950 | |
| PGD | ENSG00000142657 | -0.24 | 0.132 |  | 0.17 | 0.435 |  | -0.08 | 0.950 | |
| AKR1B1 | ENSG00000085662 | 0.34 | 0.145 |  | 0.25 | 0.435 |  | -0.10 | 0.950 | |
| SLC7A11 | ENSG00000151012 | -0.59 | 0.145 |  | -0.08 | 0.880 |  | -0.08 | 0.950 | |
| AHR | ENSG00000106546 | 0.35 | 0.145 |  | 0.31 | 0.398 |  | -0.10 | 0.950 | |
| FECH | ENSG00000066926 | -0.30 | 0.145 |  | -0.05 | 0.880 |  | -0.05 | 0.950 | |
| TALDO1 | ENSG00000177156 | -0.21 | 0.167 |  | -0.01 | 0.978 |  | -0.08 | 0.950 | |
| AKR1C1 | ENSG00000187134 | 0.43 | 0.172 |  | 0.22 | 0.650 |  | 0.04 | 0.950 | |
| MGST1 | ENSG00000008394 | -0.27 | 0.241 |  | -0.11 | 0.715 |  | -0.15 | 0.950 | |
| HMOX1 | ENSG00000100292 | 0.36 | 0.253 |  | 0.59 | 0.107 |  | 0.10 | 0.950 | |
| ME1 | ENSG00000065833 | 0.24 | 0.253 |  | 0.05 | 0.880 |  | -0.06 | 0.950 | |
| TXNRD1 | ENSG00000198431 | 0.23 | 0.253 |  | -0.06 | 0.868 |  | 0.08 | 0.950 | |
| UGDH | ENSG00000109814 | -0.29 | 0.253 |  | -0.22 | 0.581 |  | 0.12 | 0.950 | |
| PRDX6 | ENSG00000117592 | -0.15 | 0.256 |  | -0.10 | 0.650 |  | -0.05 | 0.950 | |
| GPX2 | ENSG00000176153 | 0.43 | 0.269 |  | 0.47 | 0.398 |  | 0.03 | 0.950 | |
| ACOX2 | ENSG00000168306 | 0.19 | 0.333 |  | -0.02 | 0.946 |  | 0.06 | 0.950 | |
| GCLM | ENSG00000023909 | 0.10 | 0.485 |  | -0.07 | 0.715 |  | -0.05 | 0.950 | |
| GSTP1 | ENSG00000084207 | 0.20 | 0.506 |  | 0.15 | 0.715 |  | 0.05 | 0.950 | |
| PTGR1 | ENSG00000106853 | -0.14 | 0.550 |  | -0.23 | 0.398 |  | -0.04 | 0.950 | |
| LIPH | ENSG00000163898 | -0.28 | 0.568 |  | -0.07 | 0.880 |  | -0.09 | 0.950 | |
| ACOT7 | ENSG00000097021 | 0.15 | 0.579 |  | 0.26 | 0.398 |  | -0.11 | 0.950 | |
| ACOT8 | ENSG00000101473 | -0.11 | 0.579 |  | 0.09 | 0.715 |  | -0.05 | 0.950 | |
| BLVRA | ENSG00000106605 | 0.07 | 0.579 |  | 0.10 | 0.570 |  | 0.00 | 0.982 | |
| ABCC1 | ENSG00000103222 | -0.08 | 0.579 |  | 0.19 | 0.398 |  | -0.03 | 0.950 | |
| CEBPB | ENSG00000172216 | 0.15 | 0.579 |  | 0.29 | 0.398 |  | 0.02 | 0.950 | |
| ACOX1 | ENSG00000161533 | -0.07 | 0.631 |  | -0.06 | 0.742 |  | 0.00 | 0.980 | |
| RXRA | ENSG00000186350 | 0.18 | 0.669 |  | 0.27 | 0.660 |  | -0.08 | 0.950 | |
| ABCC3 | ENSG00000108846 | -0.15 | 0.669 |  | 0.16 | 0.715 |  | 0.06 | 0.950 | |
| NQO1 | ENSG00000181019 | 0.09 | 0.691 |  | -0.04 | 0.880 |  | -0.03 | 0.950 | |
| PPARG | ENSG00000132170 | 0.11 | 0.691 |  | -0.06 | 0.871 |  | 0.10 | 0.950 | |
| PRDX1 | ENSG00000117450 | -0.05 | 0.710 |  | 0.00 | 0.978 |  | -0.04 | 0.950 | |
| ABCB6 | ENSG00000115657 | -0.05 | 0.710 |  | 0.14 | 0.435 |  | -0.03 | 0.950 | |
| FTH1 | ENSG00000167996 | 0.04 | 0.800 |  | 0.06 | 0.715 |  | -0.04 | 0.950 | |
| BLVRB | ENSG00000090013 | -0.07 | 0.806 |  | -0.01 | 0.978 |  | 0.09 | 0.950 | |
| EPHX1 | ENSG00000143819 | 0.04 | 0.839 |  | 0.11 | 0.650 |  | -0.13 | 0.950 | |
| ABCC5 | ENSG00000114770 | -0.03 | 0.861 |  | -0.09 | 0.715 |  | 0.06 | 0.950 | |
| TKT | ENSG00000163931 | -0.03 | 0.879 |  | 0.18 | 0.435 |  | 0.04 | 0.950 | |
| G6PD | ENSG00000160211 | 0.03 | 0.903 |  | 0.22 | 0.398 |  | -0.08 | 0.950 | |
| SLC6A9 | ENSG00000196517 | 0.04 | 0.903 |  | 0.29 | 0.435 |  | 0.04 | 0.950 | |
| PNPLA2 | ENSG00000177666 | -0.02 | 0.932 |  | 0.09 | 0.742 |  | 0.05 | 0.950 | |
| GPX4 | ENSG00000167468 | 0.01 | 0.932 |  | 0.00 | 0.978 |  | -0.07 | 0.950 | |
| KEAP1 | ENSG00000079999 | -0.01 | 0.962 |  | 0.11 | 0.715 |  | 0.03 | 0.950 | |
| ^a^ Students paired T-tests, adjusted by Benjamini-Hochberg for multiple testing correction | | | | | | | | | |  |
